# Combined protein and transcript single-cell RNA sequencing in human peripheral blood mononuclear cells

**DOI:** 10.1186/s12915-022-01382-4

**Published:** 2022-09-01

**Authors:** Jenifer Vallejo, Ryosuke Saigusa, Rishab Gulati, Sujit Silas Armstrong Suthahar, Vasantika Suryawanshi, Ahmad Alimadadi, Christopher P. Durant, Yanal Ghosheh, Payel Roy, Erik Ehinger, Tanyaporn Pattarabanjird, David B. Hanna, Alan L. Landay, Russell P. Tracy, Jason M. Lazar, Wendy J. Mack, Kathleen M. Weber, Adaora A. Adimora, Howard N. Hodis, Phyllis C. Tien, Igho Ofotokun, Sonya L. Heath, Avishai Shemesh, Coleen A. McNamara, Lewis L. Lanier, Catherine C. Hedrick, Robert C. Kaplan, Klaus Ley

**Affiliations:** 1 La Jolla Institute for Immunology, 9420 Athena Circle, La Jolla, CA 92037 USA; 2 Carter Immunology Center, Cardiovascular Division, Department of Medicine, University of Virginia, Charlottesville, VA USA; 3 Department of Epidemiology and Population Health, Albert Einstein College of Medicine, Bronx, NY USA; 4 Department of Internal Medicine, Rush University Medical Center, Chicago, IL USA; 5 Departments of Pathology & Laboratory Medicine and Biochemistry, University of Vermont Larner College of Medicine, Colchester, VT USA; 6 Department of Medicine, SUNY Downstate Health Sciences University, Brooklyn, NY USA; 7 Department of Medicine and Preventive Medicine, Keck School of Medicine, University of Southern California, Los Angeles, CA USA; 8 Atherosclerosis Research Unit, University of Southern California, Los Angeles, CA USA; 9 Cook County Health/Hektoen Institute of Medicine, Chicago, IL USA; 10 Department of Medicine, University of North Carolina School of Medicine, The University of North Carolina at Chapel Hill, Chapel Hill, NC USA; 11 Department of Medicine, University of California, San Francisco, San Francisco, CA USA; 12 Department of Veterans Affairs Medical Center, San Francisco, CA USA; 13 Department of Medicine, Infectious Disease Division and Grady Health Care System, Emory University School of Medicine, Atlanta, GA USA; 14 Department of Medicine, University of Alabama at Birmingham, Birmingham, AL USA; 15 Parker Institute for Cancer Immunotherapy, University of California, San Francisco, CA USA; 16 Department of Microbiology and Immunology, University of California, San Francisco, CA USA; 17 Fred Hutchinson Cancer Research Center, Public Health Sciences Division, Seattle, WA USA; 18 Department of Bioengineering, University of California San Diego, San Diego, CA USA; 19 Immunology Center of Georgia, Augusta University, Augusta, GA USA

**Keywords:** CVD, HIV, scRNA-seq, Transcriptomes, Antibodies, Human

## Abstract

**Background:**

Cryopreserved peripheral blood mononuclear cells (PBMCs) are frequently collected and provide disease- and treatment-relevant data in clinical studies. Here, we developed combined protein (40 antibodies) and transcript single-cell (sc)RNA sequencing (scRNA-seq) in PBMCs.

**Results:**

Among 31 participants in the Women’s Interagency HIV Study (WIHS), we sequenced 41,611 cells. Using Boolean gating followed by Seurat UMAPs (tool for visualizing high-dimensional data) and Louvain clustering, we identified 50 subsets among CD4+ T, CD8+ T, B, NK cells, and monocytes. This resolution was superior to flow cytometry, mass cytometry, or scRNA-seq without antibodies. Combined protein and transcript scRNA-seq allowed for the assessment of disease-related changes in transcriptomes and cell type proportions. As a proof-of-concept, we showed such differences between healthy and matched individuals living with HIV with and without cardiovascular disease.

**Conclusions:**

In conclusion, combined protein and transcript scRNA sequencing is a suitable and powerful method for clinical investigations using PBMCs.

**Supplementary Information:**

The online version contains supplementary material available at 10.1186/s12915-022-01382-4.

## Background

PBMCs are a rich source of disease- and treatment-relevant information [6, 19, 36, 48, 57, 76, 84–86]. PBMCs can be analyzed without mechanical or enzymatic dissociation, which are known to alter cell surface markers and transcriptomes [79]. PBMCs can be cryopreserved without loss of viability. At the most basic level, lymphocytes and monocyte can be distinguished by morphology using automated cell counters (blood cell counter, CBC) [7]. The current practice is to use flow cytometry of between 8 and 30 markers simultaneously [41, 46, 51, 60, 64] or even 43 markers with spectral cytometry [66]. Mass cytometry [2, 23, 71, 80] allows for analysis of up to 50 markers, but no transcriptomes [23, 61]. Single-cell RNA sequencing (scRNA-seq) allows the interrogation of expressed genes [17, 45, 53, 72, 87] and surface markers (cellular indexing of transcriptional epitope sequencing, CITE-Seq) [67, 72, 87].

In immune cells, the correlation between mRNA and surface expression of any given surface marker is, in the majority of the cases, weak [42, 67]. The correlation between mRNA and protein levels in mammalian immune cells is around 40% [69]. This is because cell surface expression is not only determined by gene expression, but also by post-translational protein modifications [43], trafficking to the cell surface, protein stability, and proteolytic modifications. The capture efficiency of mRNA is not perfect, and mRNA drop-outs further weaken the correlation between gene and surface protein [87]. Cell types in PBMCs have been defined by flow cytometry, and the surface markers of the major cell types are very well known. Yet, it is difficult to call even major cell types by scRNA-seq. For example, CD4+ T cells were not resolved from CD8+ T cells and natural killer (NK) cells [83]. To take advantage of the extensive knowledge and vast literature in flow and mass cytometry, it is necessary to assess cell surface phenotype along with transcriptomes.

Currently, only two publications report combined single-cell transcriptomes and protein (antibodies) from patients with atherosclerosis. The first one [19] includes human carotid endarterectomy specimens and matched PBMCs where 1652 PBMCs from one patient (without PBMCs from a healthy control) were analyzed by 10x Genomics 3′ and CITE-seq [53, 72], using a panel of 21 oligonucleotide-tagged antibodies. The second paper analyzed human carotid arteries from explanted hearts with 10x Genomics and a panel of 11 oligonucleotide-tagged antibodies. It is focused on smooth muscle cells and fibromyocytes rather than immune cells [81]. A recent study reported the effect of HIV infection on PBMC transcriptomes [33], focusing on acute HIV infection (before antiretroviral therapy started) and reporting PBMC transcriptomes in four patients at 8 defined time points (average of 1976 PBMC transcriptomes per participant and condition). No scRNA-seq or CITE-seq studies of PBMCs of people living with chronic HIV infection have been reported and no single-cell studies of the interaction between HIV and cardiovascular disease (CVD) are available.

Here, we report transcriptomes and cell surface phenotypes of almost 42,000 PBMCs using the targeted scRNA-seq BD Rhapsody platform [17, 45] that simultaneously provides single-cell surface phenotype (40 monoclonal antibodies, mAbs) and transcriptomes (485 immune and inflammatory transcripts) in the same cells. As a proof-of-concept, we show significant differences in cell proportions and cell transcriptomes between healthy subjects and matched subjects living with HIV or cardiovascular disease from the WIHS cardiovascular sub-study. WIHS is an ongoing multi-center, prospective, observational cohort study of women with or at risk of HIV infection. PBMCs were cryopreserved on liquid N_2_, following strict standard operating procedures that ensured preservation of cell surface phenotype, viability, and transcriptomes.

## Methods

### Study characteristics and sample selection

The Women’s Interagency HIV Study (WIHS) was initiated in 1994 at six (now expanded to ten) US locations [25, 28]. It is an ongoing prospective study of over 4000 women with or at risk of HIV infection. Recruitment in the WIHS occurred in four phases (1994–1995, 2001–2002, 2010–2012, and 2013–2015) from HIV primary care clinics, hospital-based programs, and community outreach and support groups. Briefly, the WIHS involves semi-annual follow-up visits, during which participants undergo similar detailed examinations, specimen collection, and structured interviews assessing health behaviors, medical history, and medication use. All participants provided informed consent, and each site’s Institutional Review Board approved the studies.

All participants in the current analysis were part of a vascular sub-study nested within the WIHS [25, 28, 32]. The baseline visit for the vascular sub-study occurred between 2004 and 2006, and a follow-up visit occurred on average 7 years later. Participants underwent high-resolution B-mode carotid artery ultrasound to image six locations in the right carotid artery: the near and far walls of the common carotid artery, carotid bifurcation, and internal carotid artery. A standardized protocol was used at all sites [32], and measurements of the carotid artery focal plaque, a marker of subclinical atherosclerosis, were obtained at a centralized reading center (University of Southern California). Subclinical CVD (sCVD) was defined based on the presence of one or more carotid artery lesions [32].

From the initial 1865 participants in the WIHS vascular sub-study, 32 participants were selected for scRNA-seq analysis. sCVD was defined as the presence of carotid artery focal plaque at either vascular sub-study visit to define four groups of eight participants each: HIV−, HIV+CVD−, HIV+CVD+, and HIV+CVD+ on cholesterol reduce treatment (CRT). Because we were interested in the joint relationships of HIV infection and sCVD with surface marker and RNA expression by different cell subtypes, we selected matched samples based on HIV, sCVD, and CRT (mostly statins) (Additional file 1: Fig. S1). The latter was done because we found that CRT had a major impact on monocyte transcriptomes [16]. HIV infection status was ascertained by enzyme-linked immunosorbent assay (ELISA) and confirmed by Western blot. Non-sCVD participants with self-reported coronary heart disease or current lipid-lowering therapy use were excluded. Participants were formed in quartets matched by race/ethnicity (except one quartet), age (± 5 years) at the baseline vascular sub-study (except one quartet where the age difference was more but all the women were post-menopausal), visit number, smoking history, and date of specimen collection (within 1 year).

Demographic, clinical, and laboratory variables were assessed from the same study visit using standardized protocols. The median age at the baseline study visit was 55 years, and 96% of participants were either of Black race or Hispanic ethnicity. Most (86%) reported a history of smoking. Substance use was highly prevalent, with 43% of HIV+ and 50% of HIV− participants reporting either a history of injection drug use; current use of crack, cocaine, or heroin; or alcohol use (≥14 drinks per week). Among HIV+ participants, over 80% reported use of highly active antiretroviral therapy (HAART) at the time PBMCs were obtained, and 59% reported an undetectable HIV-1 RNA level. The median CD4+ T-cell count was 585 cells/μL (IQR 382–816) in HIV+ women without sCVD and 535 cells/μL (IQR 265–792) in HIV+ women with sCVD. From now on along the text, sCVD will be referred as CVD.

### Preparation of PBMC samples for CITE-seq

To avoid batch effects, sixteen samples each were processed on the same day. PBMC tubes were thawed in a 37°C water bath and tubes filled with 8 mL of complete RPMI-1640 solution (cRPMI-1640) which contains human serum albumin, HEPES, sodium pyruvate, MEM-NEAA, penicillin-streptomycin, GlutaMax, and mercaptoethanol. Main reagents, manufacturers, and catalogue numbers are listed in Additional file 1: Table S1. The tubes were centrifuged at 400 ×g for 5 min and pellets resuspended in cold staining buffer (2 % fetal bovine serum (FBS) in phosphate-buffered saline (PBS)). Manual cell counting (trypan blue solution, 0.4%) was performed by diluting cell concentration to achieve 100–400 cells per hemocytometer count. Cells were aliquoted to a count of 1 million cells each and incubated on ice with Fc Block reagent (BD Biosciences, Additional file 1: Table S1) at a 1:20 dilution, centrifuged at 400 ×g for 5 min, resuspended in 180 μL of staining buffer, and transferred to their respective sample multiplexing kit tubes (BD Biosciences). The cells were incubated for 20 min at room temperature, transferred to 5-mL polystyrene tubes, washed 3 times, and centrifuged at 400 ×g for 5 min. The cells were resuspended in 400 μL of staining buffer and 2 μL of 0.3mM DRAQ7 and 2 μL of 2mM Calcein AM were added to each tube. The viability and cell count of each tube were determined using the BD Rhapsody scanner (Additional file 1: Table S2). Tube contents were pooled in equal proportions with total cell counts not to exceed 1 million cells. The tubes were then centrifuged at 400 ×g for 5 min and resuspended in a cocktail of 40 oligonucleotide-tagged antibodies (listed in Additional file 1: Table S3) (2 μL each antibody and 20 μL of staining buffer) on ice for 30–60 min per manufacturer’s recommendations. The tubes were then washed with 2 mL of staining buffer followed by centrifugation at 400 ×g for 5 min. This was repeated two more times for a total of 3 washes. The cells were then counted again using the scanner and loaded into Rhapsody nanowell plates (4 samples per plate).

### Library preparation

Cells were loaded at 800–1000 cells/μL into the primed plate per the BD user guide. The beads were isolated with a magnet and the supernatant removed. Reverse transcription was performed at 37 °C on a thermomixer at 1200 rpm for 20 min. Exonuclease I was incubated at 37 °C on a thermomixer at 1200 rpm for 30 min and then immediately placed on a heat block at 80 °C for 20 min. The tube was placed on ice followed by supernatant removal while beads were on a magnet. The beads were resuspended in BD bead resuspension solution. The tubes were stored at 4 °C until further processing.

Per BD’s protocol, the reagents for PCR1 including the BD Human Immune Response Panel and a custom panel of ~100 genes (Additional file 1: Table S4) were added to the beads. Samples were aliquoted into strip PCR tubes and incubated for 10 cycles according to BD’s protocol for PCR1. A double size selection was performed to remove high genomic DNA fragments by adding 0.7× volume AMPure XP SPRI beads to the PCR products. After incubation, the supernatant is recovered and transferred to a new tube followed by purifying the supernatant with an additional 100 μL of AMPure XP beads. The content was eluted off the beads using 30 μL of BD elution buffer and then transferred to a 1.5-mL tube.

### Pre-sequencing quality control (QC)

Each tube had 12 cycles of PCR performed according to BD’s user guide. The tubes were cleaned with AMPure XP beads at 0.8X for mRNA and 1.2X for sample tags. Two 200-μL washes per sample were performed during the clean-up using 80% ethanol. The cDNA was eluted off using BD elution buffer. QC and quantification was performed using Agilent TapeStation high sensitivity D1000 screen tape and Qubit double-stranded high sensitivity DNA test kit. The mRNA was then diluted, if necessary, to a concentration of 1.2–2.7 ng/μL and the antibody and sample tag libraries from PCR2 were diluted, if needed, to a concentration of 0.5–1.1 ng/μL. From each sample, 3 μL was added to a volume of 47 μL of reagents for PCR3 as described by BD’s user recommendations following the protocol and number of cycles listed, except for AbSeq, which had 9 cycles of PCR performed as determined by previous optimization. The three libraries were then cleaned with AMPure XP beads at 0.7X for AbSeq and 0.8X for sample tags. Samples were washed twice with 200 μL of 80% ethanol. The cDNA was eluted off the beads using BD’s elution buffer. Final QC and quantification was performed using TapeStation and Qubit kits and reagents.

### Sequencing

The samples were pooled and sequenced to the following nominal depth recommended by BD: AbSeq: *n* × 1000 reads per cell, where *n* is the plexity of AbSeq used; mRNA: 20,000 reads per cell; sample tags: 600 reads per cell. Thus, a total of 60,600 reads per cell were desired for sequencing on the NovaSeq. The samples and specifications for pooling and sequencing depth, along with number of cells loaded onto each plate, were optimized for S1 and S2 100 cycle kits (Illumina) with the configuration of 67 × 8 × 50 bp. Once sequencing was complete, a FASTA file was generated by BD as a reference for our AbSeq and genes we targeted with these assays. The FASTA file and FASTQ files generated by the NovaSeq were uploaded to Seven Bridges Genomics pipeline, where the data was filtered and matrices and csv files were generated. This analysis generated draft transcriptomes and surface phenotypes of 54,078 cells (496 genes, 40 antibodies). Eleven genes were not expressed, leaving 485 genes for analysis.

### Doublet removal

Based on the 4 sample tags used per plate, 8359 doublets were removed. The remaining 45,719 cells were analyzed using the Doublet Finder package on R (https://github.com/chris-mcginnis-ucsf/DoubletFinder) with the default doublet formation rate (7.5%). This removed another 3322 doublets, leaving 42,397 Cells. Finally, we removed all cells that had less than 128 (2^7^) antibody molecules sequenced. This removed 786 noisy cells, resulting in 41,611 cell transcriptomes. All antibody data were CLR (centered log-ratio) normalized and converted to log_2_ scale. All transcripts were normalized by total UMIs in each cell and scaled up to 1000.

### Thresholding

Preliminary experiments showed that each antibody had both specific and non-specific binding, as expected. To remove the non-specific signal, a threshold value separating noise from expression for each surface marker was obtained as follows (Additional file 1: Table S5). Density plots for expression of each surface marker in the main cell types (Ridgeline plots) were used to define the signal in a known negative cell population or by deconvolution of overlapping normal distributions (we used the function “normalmixEM” to deconvolute the overlapping distributions in the R package “mixtools”). The intersect of the two Gaussian distributions was then set as minimum expression threshold for the antibody, setting surface expression values below the threshold to zero. In combined protein and transcript panel single-cell sequencing, non-specific background staining is caused by incomplete Fc block and oligonucleotide-tagged antibody being trapped in the nanowell [72] as well as by incorrect titrations. Some antibodies like most antibodies to chemokine receptors have inherent background. The adjustment of antibody concentration improves signal and lowers the background [8]. Ridgeline plots of the thresholded protein expressions for each main cell type are shown in Additional file 1: Fig. S2A, which indicates how the thresholding worked on each protein expression. Based on Fig. S2A, CCR7 (CD197) antibody data were not included in the analysis.

### Clustering

Prior to clustering the data based on antibodies, we ensured that the data were batch-corrected using the Harmony package. To prepare the data for clustering, we first reduced the dimensionality of the data using UMAP (Uniform Manifold Approximation and Projection) [1] to visualize the clusters. UMAP is a manifold learning technique that helps find the latent space in which the data lies within the higher dimension space by reducing the dimensions of data. It is a dimension reduction technique used for visualization. We use Louvain clustering [3] in order to cluster the data. The parameter “resolution,” which determines the quality of clustering, is set 0.8 for B cells, 1.0 for CD4+ T cells, 1.3 for CD8+ T cells, 0.5 for classical monocytes, 0.4 for intermediate monocytes, 0.4 for nonclassical monocytes, and 0.3 for NK cells. Subclustering of each major cell type was based on all non-negative antibodies (Additional file 1: Table S6). Gates were overlaid and used in all subsequent UMAP figures (cell numbers in each cluster in Additional file 1: Table S7).

### Cluster assignment

Clustering was done just using antibodies. In CD4+ T cells, 4 of the initial clusters were further divided based on the expression of CD11c, CD56, CD25, CD127, CXCR3, and CCR2. CD8+ T cells had two clusters that were divided based on CD11c, CD16, and CXCR3 protein expression. One cluster from classical monocytes and one cluster from intermediate monocytes were further divided based on CD163 and CD152 expression, respectively. In nonclassical monocytes, one cluster showed differential expression of CD36 and CD152 expression and was divided in two. In B cells, one cluster was split because it showed differential expression of CD25 and CXCR3 within the cluster. Finally, two clusters from NK cells were split due to CD16, CD56, and CD11c expression.

### Comparing gene expression among participant types

To determine differential expression (DE) among the four types of participants, we ran Wilcoxon rank sum test in the Seurat package [73] in R with no thresholds over avg_logFC, minimum fraction of cells required in the two populations being compared, minimum number of cells and minimum number of cells expressing a feature in either group. We filtered for adjusted *p* < 0.05 and compared HIV−, HIV+CVD−, HIV+CVD+, and HIV+CVD+CRT+. From this data, dotplots were generated using ggpubr package in R. Significant genes were selected based on an adjusted *p*-value (Bonferroni corrected) threshold of < 0.05 and pct.1 (percentage of cells where the feature is detected in the first group) value > 0.2. Significantly differentially expressed genes (exact *p*-values) for each major cell types are shown in Additional file 1: Table S8.

### Comparing cell proportions

To find changes in proportions, we identified the cell numbers for each participant in each cluster (Additional file 1: Table S9). Statistical differences in cell proportions were calculated by log-odds ratio defined as *p*/(1−*p*) where *p* is the proportion of cells, followed by ANOVA and Tukey’s multiple comparison test between the four groups. For clarity, the data are presented as percentage of cells.

### Correlation analysis

We correlated each antibody to its corresponding gene(s) using Spearman rank correlation and significance (R package). For each combination of gene-antibody, we discarded cells that had values below the corresponding threshold for that antibody as well as cells with zero counts for that gene. After this filter, any gene-antibody combination that had 10 cells or less was deemed insignificant. Finally, all non-significant (*p*-value > 0.05) were designated a nominal value of zero as the Spearman rank correlation coefficient and we selected only those genes or antibodies that had at least one correlation whose coefficient ≥ 0.25 or whose coefficient ≤ −0.25. All significant non-negative correlations are reported in Additional file 1: Table S10.

### Random forest analysis

A machine learning (ML) approach was implemented to identify the genes that distinguish between disease groups. To accomplish this goal, the Random Forest (RF) model [37, 40] was trained with the normalized gene expression from 1000 randomly selected cells from each condition and variable importance scores of the genes were calculated. This procedure was repeated for 15 iterations and importance scores in each iteration were scaled to 0–100. A higher score indicated a higher power for classifying the disease groups.

## Results

### Identification of main cell types based on surface marker expression

**Fig. 1:**
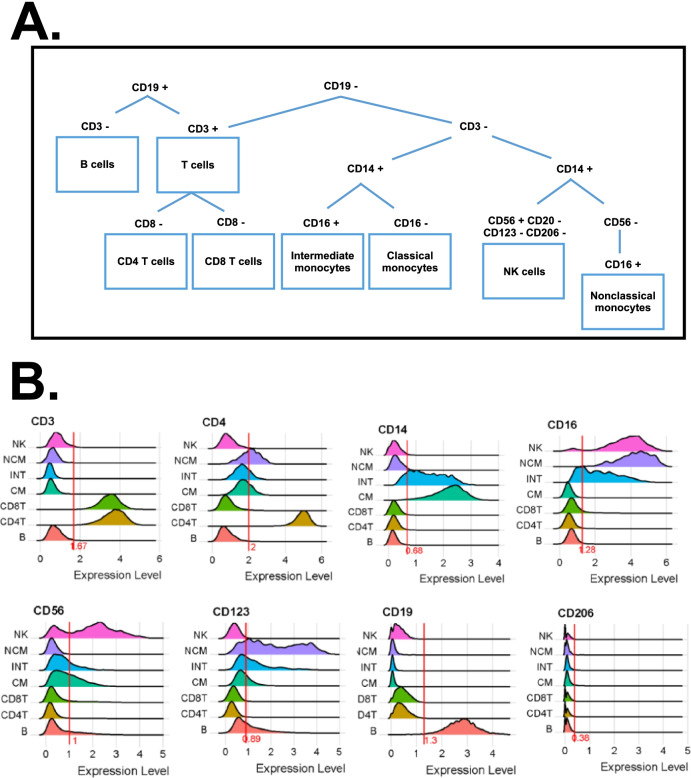

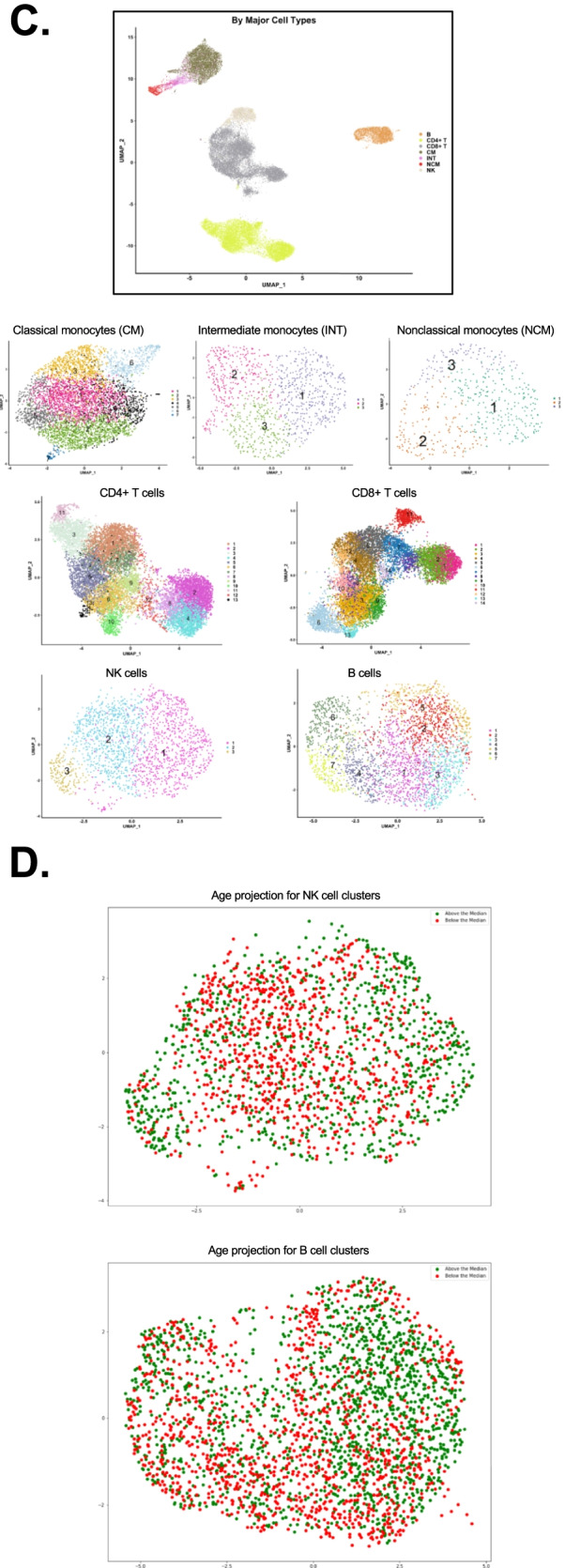
**A** Gating scheme to identify major immune cell types, **B** ridgeline plots to identify thresholds, **C** antibody-based UMAP of major cell types, and **D** age projection (above and below median age, 49 years) onto NK and B cell UMAPs. PBMCs from 32 WIHS participants were hashtagged and stained with 40 oligonucleotide-tagged antibodies. The major immune cell types were UMAP-Louvain-clustered by CD3, CD19, CD14, CD16, CD20, CD56, CD123, and CD206 surface expression. Then, each major known cell type was UMAP-Louvain-clustered separately by all non-negative surface markers. Classical monocytes (CM) formed 7 clusters, intermediate monocytes (INT) 3, and nonclassical monocytes (NCM) 3 clusters. CD4+ T cells formed 13 clusters and CD8+ T cells formed 14. NK cells formed 3 clusters and B cells formed 7 clusters. Numbers of clusters are indicated in each UMAP. Green: above median, red: below median

To identify the major known cell types (Fig. 1A), we used ridgeline plots on CD3, CD4, CD8, CD14, CD16, CD19, CD56, CD123, and CD206. This approach defines (Fig. 1B):

- B cells: CD19+ CD3−
- T cells: CD19− CD3+
- CD4+ T cells: CD4+ CD8− T cells
- CD8+ T cells: CD8+ CD4− T cells
- Monocytes (M): CD19−CD3−CD56−
- Classical (CM): CD14+CD16−
- Intermediate (INT): CD14+CD16+
- Nonclassical (NCM): CD14−CD16+CD56−
- NK cells (NK): CD4− CD56+ CD14− CD20− CD123− CD206−

This resulted in 2835 B cells, 11,019 CD4+ T cells, 10,865 CD8+ T cells, 5145 CM, 995 INT, 475 NCM, and 1801 NK cells. Each of these major cell types was then re-clustered separately, using Seurat [73] to construct UMAPs, an effective tool for visualizing high-dimensional data, with Louvain clustering (Fig. 1C). Like in flow or mass cytometry, we clustered on antibody staining only. This “preserves” the transcriptomes for investigations into disease- and treatment-related changes. Using this approach, we identified 13 CD4+ T cell subsets, 14 CD8+ T cell subsets, 7 CM subsets, 3 NCM subsets, 3 INT subsets, 7 B cell subsets, and 3 NK cell subsets (Fig. 1C). The age of each of the participants was projected onto the main cell type UMAPs to study the possible effect of this variable in the results. Similar age distribution was observed in CD4+ T cells, CD8+ cells, CM, INT, and NCM (Additional file 1: Fig. S2B). In the case of B cells and NK UMAPs, some of the clusters presented unequal age distribution, but those clusters did not show any remarkable finding in this study (Fig. 1D).

### Cell subset calling using 40 surface markers

**Fig. 2:**
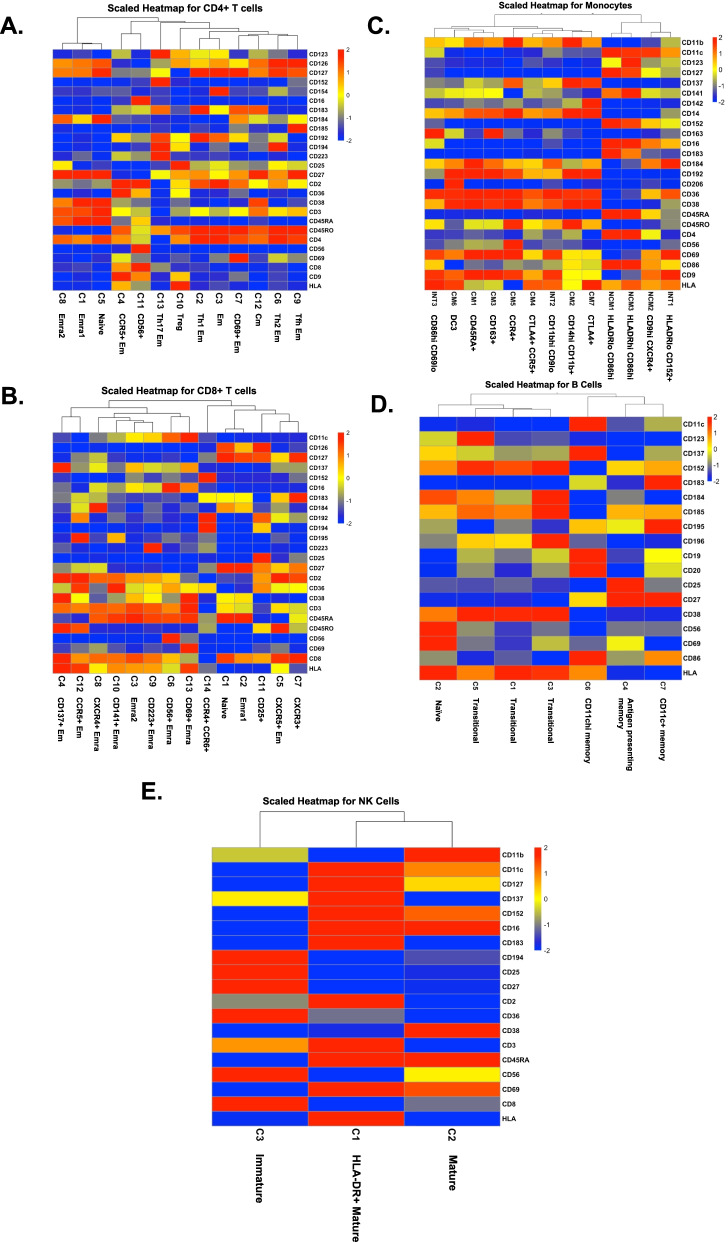
Scaled heatmaps of surface marker expression (log2 scale) in each main cell type. **A** CD4+ T cells, **B** CD8+ T cells, **C** monocytes, **D** B cells, and **E** NK cells. Immunophenotypes at the bottom. Em, effector memory; Emra, terminally differentiated effector memory; Treg, regulatory T cells; Tfh, follicular helper T cells; CM, classical monocyte; INT, intermediate monocyte; NCM, nonclassical monocyte; hi, high; lo, low. To denote each of the clusters, the letter “C” is used in CD4+ T, CD8+ T, B, and NK cells followed by the cluster number. In the case of monocytes, clusters are represented as INT, CM, or NCM followed by the cluster number. Only expressed (non-negative) markers are shown

Next, we constructed heatmaps for those antibodies that were significantly differentially expressed in at least one subset and relevant for cell calling (Fig. 2). Based on Fig. S2A, CCR7 (CD197) antibody data were not included in the analysis. This information allowed us to call all CD4+ T and CD8+ T cell subsets in accordance with the published work. Among CD4+ T cells, CD2 was expressed in almost all cells, as expected. The high affinity IL2 receptor IL2RA (CD25) was expressed in about a third of the CD4+ T cells and was strikingly high in cluster 10, which was also low for IL7 receptor (CD127), defining cluster 10 as regulatory T cells (Tregs). CD45RA and RO were mutually exclusive, separating naive and antigen-experienced CD4+ T cells. CXCR3 (CD183) identifies T-helper-1 (Th1) cells and was highly expressed in cluster 1. Cluster 9 expressed CXCR5 (CD185) as the only chemokine receptor, suggesting it may contain follicular helper (Tfh) T cells. Cluster 9 was the only cluster with high expression of CD56, suggesting a CD56+ CD4+ T cell. Based on protein information, all CD4 T cell clusters were called (Fig. 2A). Most CD8+ T cells expressed CD2. Chemokine receptors (CD183, 184, 192, 194, 195, 197) were expressed on cells in clusters 5, 7, and 8. Cluster 6 was identified as NK-like (CD56+) T cells with a CD45RA+ terminally differentiated memory (Emra) phenotype (Fig. 2B).

Among monocytes, we were able to call all the classical monocyte subsets based on published data [23, 75], related to subsets described by mass cytometry [23]. All CM were CD11b+ (Fig. 2C). There were gradients of CD9, CD69, and CD184 expression. The scavenger receptor CD36 and the chemokine receptor CCR2 (CD192) were expressed in all classical monocytes. Some markers were striking in each of the CM subsets. For example, CM cluster 7 expressed high levels of CD142 (tissue factor), which has previously been implicated in people living with HIV [68], whereas cluster 5 expressed high CD137 and cluster 3 high expression of CD163 (hemoglobin-haptoglobin receptor). INT CD14+CD16+ monocytes have been considered pro-inflammatory and are known to be increased in people with HIV [24] and with CVD [62, 74]. All INT monocytes highly expressed the activation molecules CD69, CD9, and CD36 (Fig. 2C). Since INT subsets have not been described before, we propose a provisional naming suggestion (Fig. 2C) based on their most remarkable expressed markers. NCM formed 3 clusters. Strikingly, the expression of CD9 and CD36 was limited to cluster 2, suggesting that this cluster corresponds to the previously described CD9+CD36+ NCM [23]. CD11c, CD86, CD141, and CD152 were expressed in all NCMs (Fig. 2C).

Using 18 surface antibodies to subtype B cells obtained from women with coronary artery diseases, B cells were subtyped into 7 clusters (Fig. 2D) with 3 clusters of CD27+ B cells and 4 clusters of CD27− B cells. CD27 is known as a conventional memory B cell marker [31]. Therefore, clusters 4, 6, and 7 were classified as memory B cell populations. Clusters 6 and 7 were notable for high CD11c expression. CD11c+ memory B cells have been shown to be precursors of antibody secreting cells [22]. Cluster 4 was remarkable for CD25+, and CD25+ B cells are known as antigen presenting B cells [5]. Clusters 1, 2, 3, and 5 were CD27− B cells, likely a combination of naïve B cells and transitional B cells.

Among NK cells, we found 3 clusters. Cluster 1 contained HLADR+ mature NK cells with CD56-CD16^high^ expression, an NK cell subset known to be elevated in chronic HIV infection [29]. Cluster 2 of NK cells was mature (CD56^dim^/CD16+). Cluster 3 contained immature (CD56^bright^CD16−) NK cells (Fig. 2E).

### Changes in PBMC subset abundance by disease or treatment

**Fig. 3:**
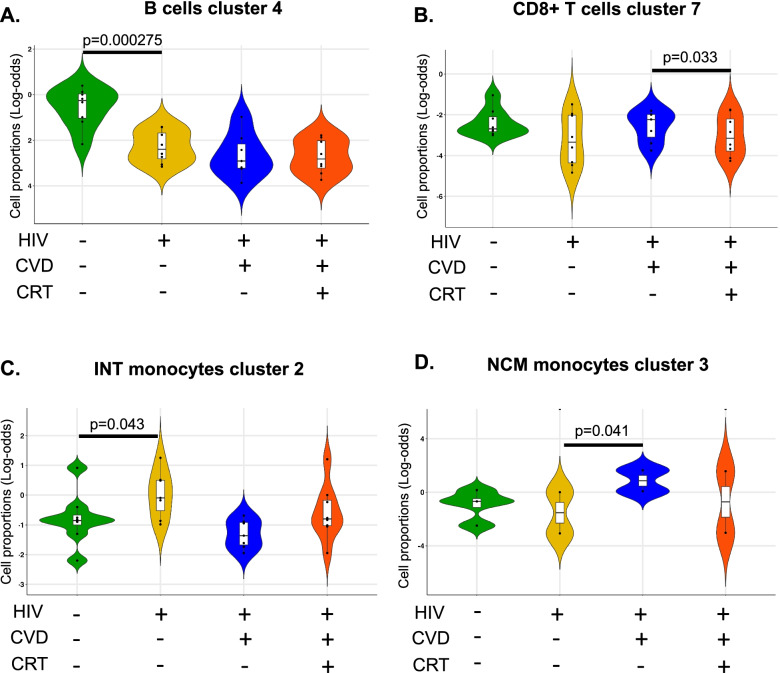
Cell proportions in women with HIV, CVD, both, or neither. HIV−CVD− (green), HIV+CVD− (yellow), HIV+CVD+ (blue), and HIV+CVD+ CRT+ (red), from left to right. Eight samples per group except 7 for HIV+CVD+. Proportions of cells in each cluster calculated as percentage of the parent cell type as indicated in the title of each panel. **A** B cell cluster 4, **B** CD8+ T cell cluster 7, **C** INT monocyte cluster 2, and **D** NCM cluster 3. Clusters with significant differences (*p*-value) in cell proportions (calculated by log odds ratio (*p*/(1−*p*)), followed by ANOVA and Tukey’s multiple comparison test), are shown with individual points as well as means and standard error of the mean (SEM). INT, intermediate monocytes; NCM, nonclassical monocytes; CVD, cardiovascular disease; CRT, cholesterol-reducing treatment

Based on this data, it is possible to address shifts in cell proportion based on disease or treatment: HIV effect, comparing HIV− vs HIV+; the cardiovascular disease effect in women living with HIV, comparing HIV+ vs HIV+CVD+; and the effect of cholesterol reduction treatment, comparing HIV+ CVD+CRT− vs HIV+ CVD+ CRT+. We found significant differences in cell proportions (*p*) (calculated by log odds ratio, *p*/(1−*p*) followed by ANOVA and Tukey’s multiple comparison test) in one intermediate monocyte subsets, one nonclassical monocyte subset, one CD8+ T cell, and one B cell subset (Fig. 3). Among B cells, antigen presenting memory B cells (Fig. 3A) were severely lower in all WIHS participants with HIV with or without subclinical CVD. Within CD8+ T cells, cluster 7 (CXCR3+ CD8+ T cells) was reduced in patients with HIV and CVD with CRT treatment compared to those without cholesterol-reducing treatment (Fig. 3B). Strikingly, two subsets of monocytes showed significantly different abundances. Cluster 2 from intermediate monocytes (CD11bhi CD9lo) (Fig. 3C) was elevated in patients with HIV and cluster 3 from nonclassical monocytes (CD9hi CXCR4+) (Fig. 3D) was significantly elevated in WIHS participants living with HIV with subclinical CVD.

### Differential gene expression in each of the clusters

**Fig. 4:**
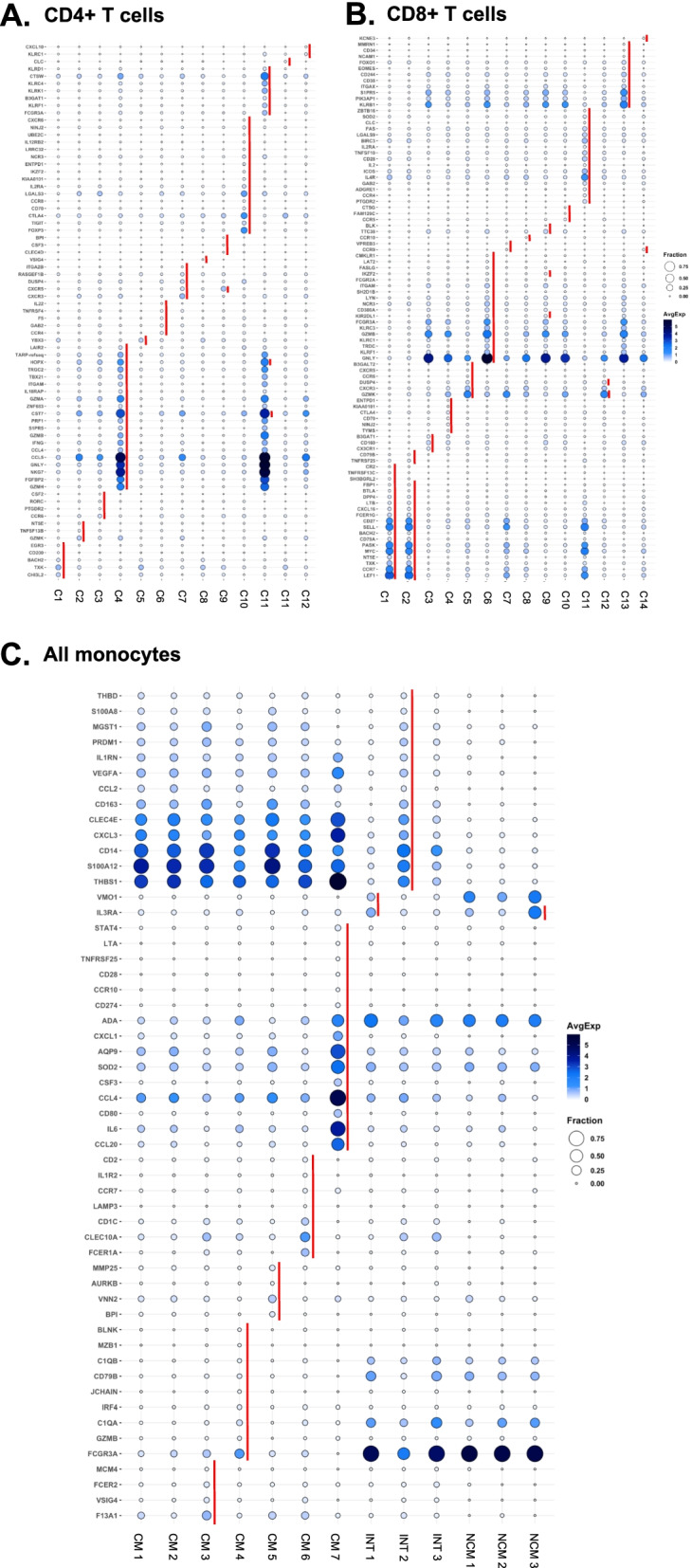
Significantly differentially expressed genes of cells in each cluster. The expression of 485 transcripts was determined by targeted amplification (BD Rhapsody system). Significant genes defined as adjusted *p* < 0.05 and log2 fold change > 0. Dot plot: fraction of cells in cluster expressing each gene shown by size of circle and level of expression shown from white (=0) to dark blue (=max, log2 scale). Red bars indicate genes that were significantly higher in one cluster compared to all other clusters of the parent cell type. There were no DEGs in NK or B cell clusters. **A** CD4+ T cells, **B** CD8+ T cells, and **C** monocytes. CM, classical monocytes; INT, intermediate monocytes; NCM, nonclassical monocytes. To denote each of the clusters, the letter “C” is used in CD4+ T and CD8+ T followed by the cluster number. In the case of monocytes, clusters are represented as INT, CM, or NCM followed by the cluster number

Since the transcriptomic information was not used for UMAPs and clustering shown in Fig. 1C, we were able to run unbiased gene expression patterns in each cell subset against all other clusters within the same cell type. We filtered for genes that were significantly differentially expressed in at least one of the subsets (Fig. 4, Additional file 1: Table S8). This analysis revealed gene signatures for most subsets and confirmed the identity of the cell clusters identified by CITE-seq (Fig. 2) and expanded phenotype information. Cluster 4 CD36+ effector memory CD4+ T cells overexpressed *GNLY*, *GZMA*, *GZMH*, *NKG7*, and *FGFBP2*, which are known as effector memory (Em) signature genes. Cluster 10 expressed regulatory T cell (Treg) signature genes including *FoxP3*, *TIGIT*, *CTLA4*, and *LGALS3*. Cluster 9 Tfh Em CD4+ T cells expressed CXCR5, the classical chemokine receptor characteristic for Tfh cells [13]. CD56+ CD4+ T cells (cluster 11) expressed NK signatures such as *HOPX*, *CTSW*, *KLRC4*, and *KLRK* (Fig. 4A). Within CD8+ T cells, cluster 2 (naïve) overexpressed *CCR7*, *SELL*, or *LEF1*, representative genes of naïve cells. NK-like phenotype genes such as *GNLY*, *KLRF1*, *GZMB*, and *FCGR3A* were expressed in cluster 6 (CD56+ EMRA) (Fig. 4B). CTLA4+ CM (cluster 7) expressed *CCL20*, *SOD2*, or *AQP9*, classical monocyte markers (Fig. 4C). No significant differences were found for B cells and NK cells.

### Transcriptomes shift with HIV, CVD, and cholesterol control

**Fig. 5:**
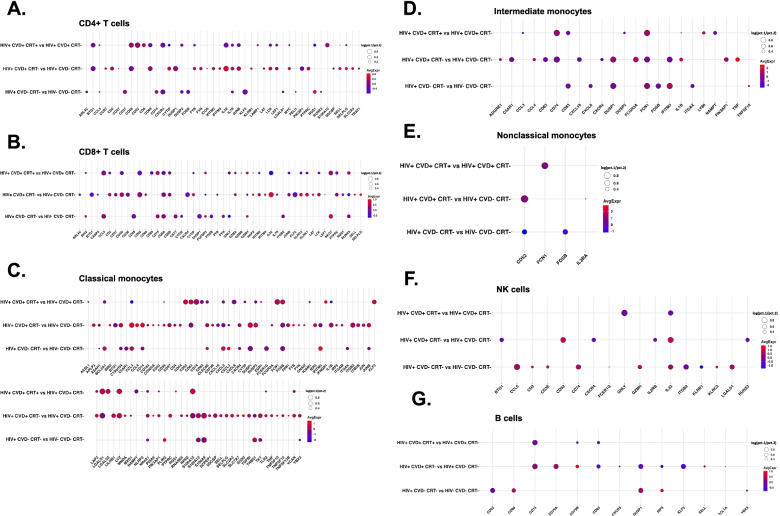
Dotplots of differentially expressed genes (DEGs) for disease types. Dotplots of DEGs between HIV+CVD−CRT− vs HIV−CVD−CRT−, between HIV+CVD+CRT− vs HIV+CVD−CRT−, and between HIV+CVD+CRT+ vs HIV+CVD+CRT− in each cell type (**A–G**). The thresholds set for the plots were adjusted *p*-value <0.05, avg. log2FC>0 or <0, and pct.1 > 0.2. The size of dots represents log(pct.1/pct.2), where pct.1 is the proportion of cells in the first group expressing each gene and pct.2 is the proportion of cells in the second group expressing each gene

We hypothesized blood immune cell transcriptomes change with disease state. To test this possibility, we constructed dotplots for each of the disease status and compared HIV− with HIV+, HIV+ with HIV+ with CVD, and HIV+CVD+ with HIV+ CVD+ with CRT treatment (Fig. 5, Additional file 1: Fig. S3, Additional file 2: Data S1). Many genes in CD4+ and CD8+ T cell subsets showed significant differences. Some genes in monocyte and NK cell subsets showed significant differences. In CD4+ T, CD8+ T, and NK cells (Fig. 5A, B, F), *IL-32* was highly significantly increased by CVD, but not in CVD+ women on CRT. IL-32 is an inflammatory cytokine that is known to be important in CVD [14, 34]. The transcription factor *JUNB* and the lymphocyte-specific protein tyrosine kinase *LCK* were upregulated in both, CD4+ T and CD8+ T cells from women with CVD, and reduced in the presence of CRT. *JUMB* promotes the development of inflammatory Th17 cells and restricts flexibility towards alternative effector and regulatory programs [9]. *LCK* is a key molecule in the activation of the TCR signaling and T cells. Several killer cell lectin receptors (*KLRC4* and *KLRK1*) were also significantly upregulated in CVD, but downregulated in the presence of CRT in CD8+ T cells. *RUNX* is reduced in CVD but increased in women on CRT [35].

*DUSP1* was highly overexpressed in classical monocytes of women with CVD (Fig. 5C). *DUSP1* oxidation prolongs MAPK activation, ultimately resulting in enhanced inflammatory responses [30]. In addition to *CCL3*, *CCL4*, and *DUSP2*, *IL1B*, known to be highly relevant in atherosclerosis, was highly upregulated in CM of HIV+CVD+ women. *TNFSF10* (TRAIL), *TNFSF13* (APRIL), and *TNFSF13B* (BAFF), important B cell regulators, were upregulated in CM from women with CVD. The Toll-like receptor *TLR2*, which is known to be involved in atherosclerosis [15, 49, 55], was upregulated by CVD. In intermediate monocytes (Fig. 5D), *CCL4*, *TNF*, *IL1B*, *FCGR3A*, and *PIK3AP1* were associated with CVD in the women that did not receive CRT whereas *FCN1* was increased in NCM from women with CRT (Fig. 5E). In nonclassical monocytes, *CD52* was downregulated in women with HIV, consistent with a previous report [77]. CD74 in B cells (Fig. 5G) was slightly downregulated in CVD women on CRT compared to CVD. DUSP1 in B cells was downregulated in HIV+CVD+ women compared to HIV+ CVD−. The clusters with genes that passed the filtering process (*p*-value <0.05, avg.Log2FC>0 or <0, and pct.1 > 0.2) from each of the main cell types are presented in Fig. S3.

### Random Forest analysis to identify genes that distinguish between disease groups

**Fig. 6:**
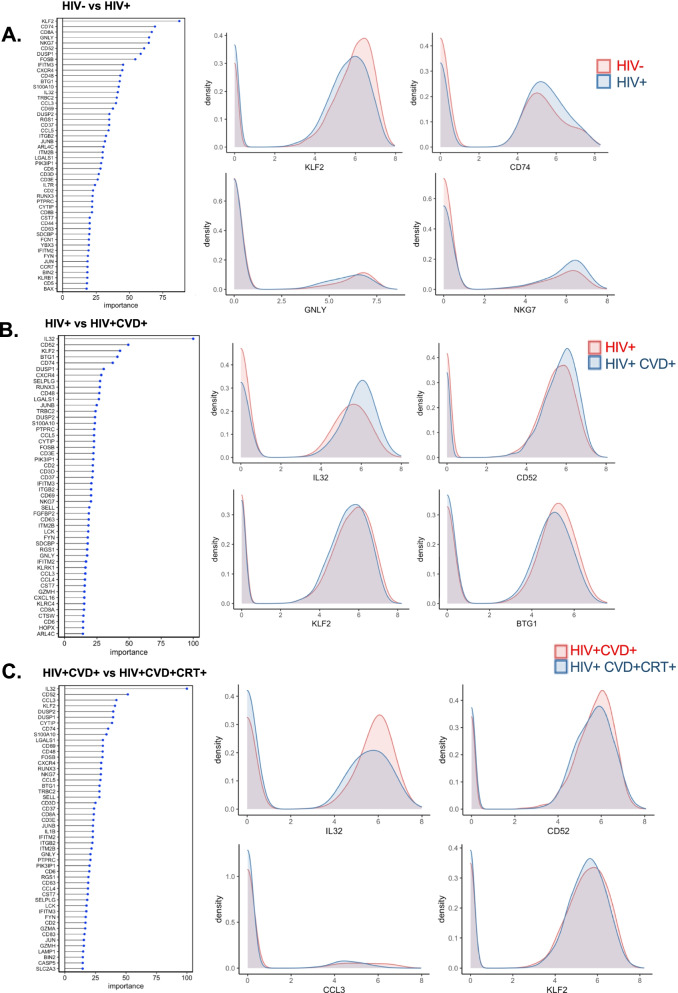
Machine learning analysis using the Random Forest model. Line with dot plots showing the feature importance of each gene in the comparison between **A** non-HIV vs HIV, **B** HIV+CVD− vs HIV+CVD+, and **C** HIV+CVD+CRT− vs HIV+CVD+CRT+. On the right, representative density plots are shown: **A** red, HIV−; blue, HIV+. **B** red, HIV+; blue, HIV+CVD+ and **C** red, HIV+CVD+; blue, HIV+CVD+CRT+

We used the Random Forest Machine learning approach to identify the genes with the highest capability to distinguish between disease groups (Additional file 2: Data S2). We showed the top 50 ranked genes to separate HIV vs healthy (Fig. 6A), HIV vs HIV+CVD+ (Fig. 6B), and HIV+CVD+ vs HIV+CVD+CRT+ (Fig. 6C), with the importance ranked between 0 and 100. The histograms in Fig. 6A–C show overlaid ridgeline plots for key genes. *KLF2* was the most important gene to separate HIV from non-HIV participants. Both in CVD and in CVD+CRT+ in HIV, *IL32*, and *CD52* were highly ranked (Fig. 6B, C). Interestingly, these genes were upregulated in CVD and conversely were downregulated in CVD+CRT+. Many of the genes that were important in distinguishing between women with and without CVD, including *DUSP1*, *DUSP2*, *CCL5*, and *LGALS1*, were regulated in the opposite direction by CRT.

## Discussion

In immunology, surface markers are widely used to define and distinguish cell types [11, 71, 80]. Flow cytometry is the discipline-defining method of immunology [60]. Similar to flow cytometry, in CyTOF, single-cell suspensions are stained with antibody panels to detect cellular antigens. Unlike CyTOF, scRNA-seq allows the detection of single-cell transcriptomes. Since the correlation between cell surface protein and mRNA expression is weak in immune cells [42], the transcriptome provides a valuable additional dimension. scRNA-seq without surface phenotype information has led to much frustration in the field, because the expression of many genes encoding well-known surface markers remains undetected in scRNA-seq [10, 44, 80]. It is still difficult to call cell types based on gene expression data alone, which emphasizes the need for cell surface phenotypes in addition to transcriptomes. Here, we correlated gene expression with cell surface expression for 41 pairs of genes and proteins. CD74 protein expression was well correlated with the expression of both the *CD74* and the *HLA-DRA* genes. CD4 and CD16 surface and gene expression were reasonably well correlated across all cell types. A few other genes including *CD14*, *CD16*, IL-3 receptor (*CD123*), and *CD27* were somewhat correlated with the surface expression of their proteins in some cell types. For most markers, we confirm weak correlations (Additional file 1: Table S10) [42, 67, 69], which illustrates the value of monitoring cell surface phenotype in scRNA-seq.

PBMCs can be analyzed without mechanical or enzymatic dissociation, which are known to alter cell surface markers and transcriptomes [79. ]. PBMCs are attractive for single-cell RNA sequencing (scRNA-seq) studies, because they are available in many clinical studies of specific populations with defined diseases and outcomes. The participants sampled for the present study were part of a sub-study nested within the WIHS [25, 28, 32], which provided detailed information on subclinical atherosclerosis. Participants underwent high-resolution B-mode carotid artery ultrasound to image six locations in the right carotid artery [28]. Although our study is not definitive, it is suggestive of significant changes in cell proportions and transcriptomes in subjects with cardiovascular disease.

scRNA-seq has been applied to human PBMCs in diseases including cancers [6, 84–86], inflammatory bowel disease [48, 76], and autoimmune disease [36, 57], as well as atherosclerosis [18, 19, 81]. The foundational paper for the 10x Genomics drop-Seq method [87] demonstrated the feasibility of using scRNA-seq on PBMCs. Other studies reported scPBMC transcriptomes in colorectal cancer [85], γδ T cells [54], liver cancer [86], in vitro *Salmonella* infection [4], and memory T cells [45]. Two publications have reported combined single-cell transcriptomes and proteomics from patients with atherosclerosis. Fernandez et al. [19] ran antibody sequencing in only one human plaque, but its power was revealed by identifying five distinct macrophage clusters. Wirka et al. [81] revealed that the process of smooth muscle cell phenotypic modulation in vivo can be altered by the expression of *Tcf21*, a gene causally associated with reduced risk of coronary artery disease. The loss of *Tcf21* results in fewer fibromyocytes in the lesions and the protective fibrous cap [81].

In the current study, four clusters showed significantly different abundance of cells in the four groups of participants. One of them is an intermediate monocyte subset, which underscores the extraordinary importance of this cell type in chronic HIV infection [26, 47]. Intermediate monocyte numbers have previously been found increased in non-HIV subjects with peripheral artery occlusive disease [78] and significantly predicted cardiovascular events [27, 62, 63]. A nonclassical monocyte cluster was increased in CVD, which was reported previously [20, 39].

CD8+ T cells are abundant in atherosclerotic plaques in humans [21] and they are found in higher numbers than CD4+ cells [19, 21, 65]. In advanced human lesions, CD8+ T cells are mostly found in fibrous cap areas [52]. Cluster 7 of CD8 + T cells (CXCR3+) was reduced in women on CRT. CD8+ T cells express higher levels of CXCR3 in patients with symptomatic atherosclerosis [19]. We found *IL-32* highly expressed in most T and NK cell clusters in CVD. IL-32 is a 27-kDa cytokine expressed in T cells and monocytes that is secreted after apoptosis [50]. It is an inflammatory cytokine that drives IL-1β, clinically important in CVD [59], TNF, IL-6, and IL-8 expression [14, 34, 50]. IL-32 activates the leukocyte surface protease PR3, which in turn triggers the G-protein-coupled receptor PAR2 [50] and is known to be important in viral infections [38, 50, 56, 70]. IL32 was upregulated in CVD and was downregulated in women with CVD and cholesterol reduce treatment. Since IL-32 appears to be CVD-specific, we advocate for future prospective studies in larger cohorts to determine whether IL32 mRNA is a useful biomarker.

*KLF2* was the most important gene to separate HIV from non-HIV participants. Some previous studies showed that KLF2 is related to HIV infection [12, 58]. This gene and some others including *CD74* and *CD52* were also important to separate CVD from non-CVD in HIV, and CVD+CRT+ from CVD+, suggesting that these genes may be related to HIV and CVD. In fact, it has been reported that anti-CD52 antibodies might be effective in HIV individuals on antiretroviral therapy [77] as well as a potential diagnostic value evaluating antiretroviral efficiency [82]. The interaction between HIV and CVD observed here is novel.

Our discovery study will encourage prospective epidemiological studies to address which PBMC subsets and transcriptomes are best suited as clinical biomarkers to stratify risk and guide treatment in subjects with coronary or peripheral artery disease. The current findings have some limitations. They need to be extended to men (the current data is based on women) and other races and ethnicities (the current data is based on mostly African American and Hispanic women). Studies of CVD in non-smokers are also needed (the current data is based on smokers), and the age range needs to be broadened.

## Conclusions

In conclusion, we demonstrate the utility of scRNA-seq with cell surface phenotype assessment in the same cells. The identification of 50 distinct clusters of CD4+ T and CD8+ T cells, B cells, NK cells, and monocytes helps to gain a deeper understanding of PBMCs, a rich and readily accessible source of biological and clinical information. The discovery of subsets of intermediate monocytes calls for identifying such subsets in model organisms to test their function in vivo.

## Supplementary Information

**Additional file 1: ** Supplementary Figures and Tables. **Figure S1.** (Related to Figure 1). Design of the study comparison between study participants. **Figure S2.** (Related to Figure 1). (**A**) Ridgeline plots of the unthresholded expressions of all the 40 surface markers for each main cell types. (**B**) Age projection (below and above median) onto CM, INT, NCM, CD4+ T cells and CD8+ T cell UMAPs. **Figure S3.** (Related to Figure 5). Dotplots of DEG for disease types for subsets. **Table S1.** Reagents. **Table S2.** The cell viability of each frozen PBMC tube. **Table S3.** List of 40 titrated oligonucleotide-tagged monoclonal antibodies. **Table S4.** List of selected genes included in the custom panel. **Table S5.** Threshold values of each antibody. **Table S6.** Antibodies not used for cell clustering. **Table S7.** Number of Cells in each of the clusters. **Table S8.** Significantly differentially expressed genes for each cell type (A-E). **Table S9.** Number of Cells for each participant. **Table S10.** Non-negative Spearman correlation between antibody and gene in each cell type.

**Additional file 2: Data S1.** (separate excel file). Data underlying Figure 5, dotplots of DEGs for disease types.

**Additional file 3: Data S2.** (separate excel file). Data underlying Figure 6, random forest model.

## Data Availability

All data are available in the main text or the supplementary materials. The datasets presented in this study can be found in online repositories. The name of the repository and accession number for the data reported in this paper is Gene Expression Omnibus (GEO), GSE205320 [88].

## Abbreviations

CBC: Blood cell counter
CITE-seq: Cellular indexing of transcriptional epitope sequencing
CLR: Centered log-ratio
CM: Classical monocytes
CRT: Cholesterol reduce treatment
CVD: Cardiovascular disease
DE: Differential expression
ELISA: Enzyme-linked immunosorbent assay
Em: Effector memory
Emra: Terminally differentiated memory
FBS: Fetal bovine serum
HAART: Highly active antiretroviral therapy
INT: Intermediate
M: Monocytes
mAbs: Monoclonal antibodies
ML: Machine learning
NCM: Nonclassical monocyte
NK: Natural killer
PBMCs: Peripheral blood mononuclear cells
PBS: Phosphate-buffered saline
QC: Quality control
RF: Random forest
scRNA-seq: Single-cell RNA sequencing
sCVD: Subclinical CVD
Tfh: Follicular helper T cells
Tregs: Regulatory T cells
UMAP: Uniform manifold approximation and projection
WIHS: Women’s Interagency HIV Study
