# Author Correction: Combined protein and transcript single-cell RNA sequencing in human peripheral blood mononuclear cells

**DOI:** 10.1186/s12915-022-01440-x

**Published:** 2022-10-20

**Authors:** Jenifer Vallejo, Ryosuke Saigusa, Rishab Gulati, Sujit Silas Armstrong Suthahar, Vasantika Suryawanshi, Ahmad Alimadadi, Christopher P. Durant, Yanal Ghosheh, Payel Roy, Erik Ehinger, Tanyaporn Pattarabanjird, David B. Hanna, Alan L. Landay, Russell P. Tracy, Jason M. Lazar, Wendy J. Mack, Kathleen M. Weber, Adaora A. Adimora, Howard N. Hodis, Phyllis C. Tien, Igho Ofotokun, Sonya L. Heath, Avishai Shemesh, Coleen A. McNamara, Lewis L. Lanier, Catherine C. Hedrick, Robert C. Kaplan, Klaus Ley

**Affiliations:** 1 La Jolla Institute for Immunology, 9420 Athena Circle, La Jolla, CA 92037 USA; 2 Carter Immunology Center, Cardiovascular Division, Department of Medicine, University of Virginia, Charlottesville, VA USA; 3 Department of Epidemiology and Population Health, Albert Einstein College of Medicine, Bronx, NY USA; 4 Department of Internal Medicine, Rush University Medical Center, Chicago, IL USA; 5 Departments of Pathology & Laboratory Medicine and Biochemistry, University of Vermont Larner College of Medicine, Colchester, VT USA; 6 Department of Medicine, SUNY Downstate Health Sciences University, Brooklyn, NY USA; 7 Department of Medicine and Preventive Medicine, Keck School of Medicine, University of Southern California, Los Angeles, CA USA; 8 Atherosclerosis Research Unit, University of Southern California, Los Angeles, CA USA; 9 Cook County Health/Hektoen Institute of Medicine, Chicago, IL USA; 10 Department of Medicine, University of North Carolina School of Medicine, The University of North Carolina at Chapel Hill, Chapel Hill, NC USA; 11 Department of Medicine, University of California, San Francisco, San Francisco, CA USA; 12 Department of Veterans Affairs Medical Center, San Francisco, CA USA; 13 Department of Medicine, Infectious Disease Division and Grady Health Care System, Emory University School of Medicine, Atlanta, GA USA; 14 Department of Medicine, University of Alabama at Birmingham, Birmingham, AL USA; 15 Parker Institute for Cancer Immunotherapy, University of California, San Francisco, CA USA; 16 Department of Microbiology and Immunology, University of California, San Francisco, CA USA; 17 Fred Hutchinson Cancer Research Center, Public Health Sciences Division, Seattle, WA USA; 18 Department of Bioengineering, University of California San Diego, San Diego, CA USA; 19 Immunology Center of Georgia, Augusta University, Augusta, GA USA

**Correction: BMC Biol 20, 193 (2022)**

**https://doi.org/10.1186/s12915-022-01382-4**

The original article [1] contained significant errors in Fig 1A which necessitated correction; the figure has since been updated to the corrected version.
