# Supplementary material for: Combined protein and transcript single-cell RNA sequencing in human peripheral blood mononuclear cells
Source: BMC Biol. 2022 Sep 1;20:193. doi: 10.1186/s12915-022-01382-4 (PMC9434837; doi:10.1186/s12915-022-01382-4)
Supplement: Supplementary file 1 — Additional file 1: Supplementary Figures and Tables. Figure S1. (Related to Figure 1). Design of the study comparison between study participants. Figure S2. (Related to Figure 1). (A) Ridgeline plots of the unthresholded expressions of all the 40 surface markers for each main cell types. (B) Age projection (below and above median) onto CM, INT, NCM, CD4+ T cells and CD8+ T cell UMAPs. Figure S3. (Related to Figure 5). Dotplots of DEG for disease types for subsets. Table S1. Reagents. Table S2. The cell viability of each frozen PBMC tube. Table S3. List of 40 titrated oligonucleotide-tagged monoclonal antibodies. Table S4. List of selected genes included in the custom panel. Table S5. Threshold values of each antibody. Table S6. Antibodies not used for cell clustering. Table S7. Number of Cells in each of the clusters. Table S8. Significantly differentially expressed genes for each cell type (A-E). Table S9. Number of Cells for each participant. Table S10. Non-negative Spearman correlation between antibody and gene in each cell type. [file 12915_2022_1382_MOESM1_ESM.docx]

Journal: BMC Biology (BMCB-D-22-00576)

Supplementary Materials for

**Title (BMCB-D-22-00576)**

Combined protein and transcript single cell RNA sequencing in human peripheral blood mononuclear cells

**Authors**

Jenifer Vallejo,^1#^ Ryosuke Saigusa,^1#^ Rishab Gulati,^1^ Sujit Silas Armstrong Suthahar,^1^ Vasantika Suryawanshi,^1^ Ahmad Alimadadi,^1^ Christopher P. Durant,^1^ Yanal Ghosheh,^1^ Payel Roy,^1^ Erik Ehinger,^1^ Tanyaporn Pattarabanjird,^2^ David B. Hanna,^3^ Alan L. Landay,^4^ Russell P. Tracy,^5^ Jason M. Lazar,^6^ Wendy J. Mack,^7,8^ Kathleen M. Weber,^9^ Adaora A. Adimora,^10^ Howard N. Hodis,^7,8^ Phyllis C. Tien,^11^ Igho Ofotokun,^12^ Sonya L. Heath,^13^ Avishai Shemesh,^14^ Coleen A. McNamara,^2^ Lewis L. Lanier,^14^ Catherine C. Hedrick,^1^ Robert C. Kaplan,^3,15^ Klaus Ley.^1,16*^

**Affiliations**

^1^ La Jolla Institute for Immunology, La Jolla, CA, USA.

^2^ Carter Immunology Center, Cardiovascular Division, Department of Medicine, University of Virginia, Charlottesville, VA, USA.

^3^ Albert Einstein College of Medicine, Department of Epidemiology and Population Health, Bronx, NY, USA.

^4^ Rush University Medical Center, Department of Internal Medicine, Chicago, IL, USA.

^5^ University of Vermont Larner College of Medicine, Departments of Pathology & Laboratory Medicine and Biochemistry, Colchester, VT, USA.

^6^ SUNY Downstate Health Sciences University, Department of Medicine, Brooklyn, NY, USA.

^7^ Keck School of Medicine, University of Southern California, Department of Medicine and Preventive Medicine, Los Angeles, CA, USA.

^8^ Atherosclerosis Research Unit, University of Southern California, Los Angeles, CA, USA.

^9^ Cook County Health/Hektoen Institute of Medicine, Chicago, IL, USA.

^10^ Department of Medicine, University of North Carolina School of Medicine, The University of North Carolina at Chapel Hill, Chapel Hill, NC, USA.

^11^ Department of Medicine, University of California, San Francisco, San Francisco, CA and Department of Veterans Affairs Medical Center, San Francisco, CA, USA.

^12^ Emory University School of Medicine, Department of Medicine, Infectious Disease Division and Grady Health Care System, Atlanta, GA, USA.

^13^ University of Alabama at Birmingham, Department of Medicine, Birmingham, AL, USA.

^14^ Parker Institute for Cancer Immunotherapy, University of California, San Francisco, CA, USA; Department of Microbiology and Immunology, University of California, San Francisco, CA, USA.

^15^ Fred Hutchinson Cancer Research Center, Public Health Sciences Division, Seattle, WA, USA.

^16^ Department of Bioengineering, University of California San Diego, San Diego, CA, USA.

^#^ Contributed equally to this work

Corresponding Author

Klaus Ley, MD

La Jolla Institute for Immunology

9420 Athena Circle

La Jolla, CA 92037, USA

(858) 752-6661 (tel)

(858) 752-6985 (fax)

[klaus@lji.org](mailto:klaus@lji.org)

Effective August 1^st^ 2022:

Klaus Ley, M.D.

Co-Director, Immunology Center of Georgia (IMMCG)

GRA Bradley Turner Eminent Scholar Chair in Immunology

Professor of Physiology

Augusta University

Immunology Center of Georgia

1410 Laney Walker Blvd, CN4315

Augusta, GA 30912

email kley@augusta.edu

**This file (Additional file 1) includes:**

Figures S1 to S3

Tables S1 to S10

**Other Supplementary Materials for this manuscript include the following:**

Additional file 2: Data S1

Additional file 2: Data S2

Figure S1.


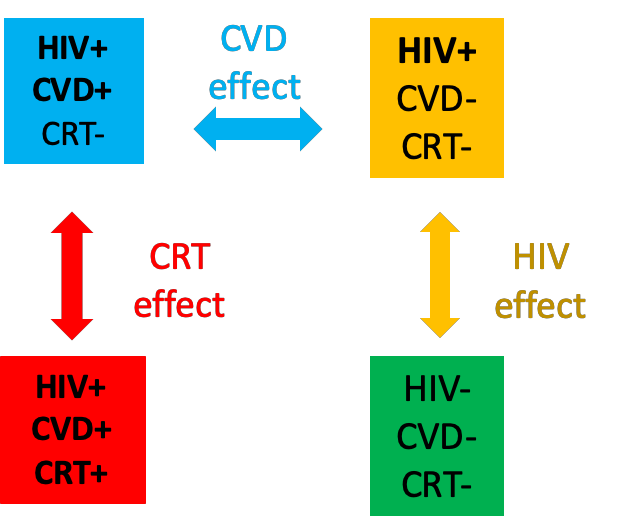


**Figure S1. (related to Figure 1). Design of the study comparison between study participants.** Three different comparisons are included in this study. The HIV effect (yellow), comparing HIV- vs HIV+, the cardiovascular disease effect in women living with HIV (blue), comparing HIV+ vs HIV+CVD+ and the effect of cholesterol reduction treatment (red) (the CRT effect, comparing HIV+ CVD+CRT- vs HIV+ CVD+ CRT+). CVD, cardiovascular disease; CRT, cholesterol reduce treatment.

Fig. S2.


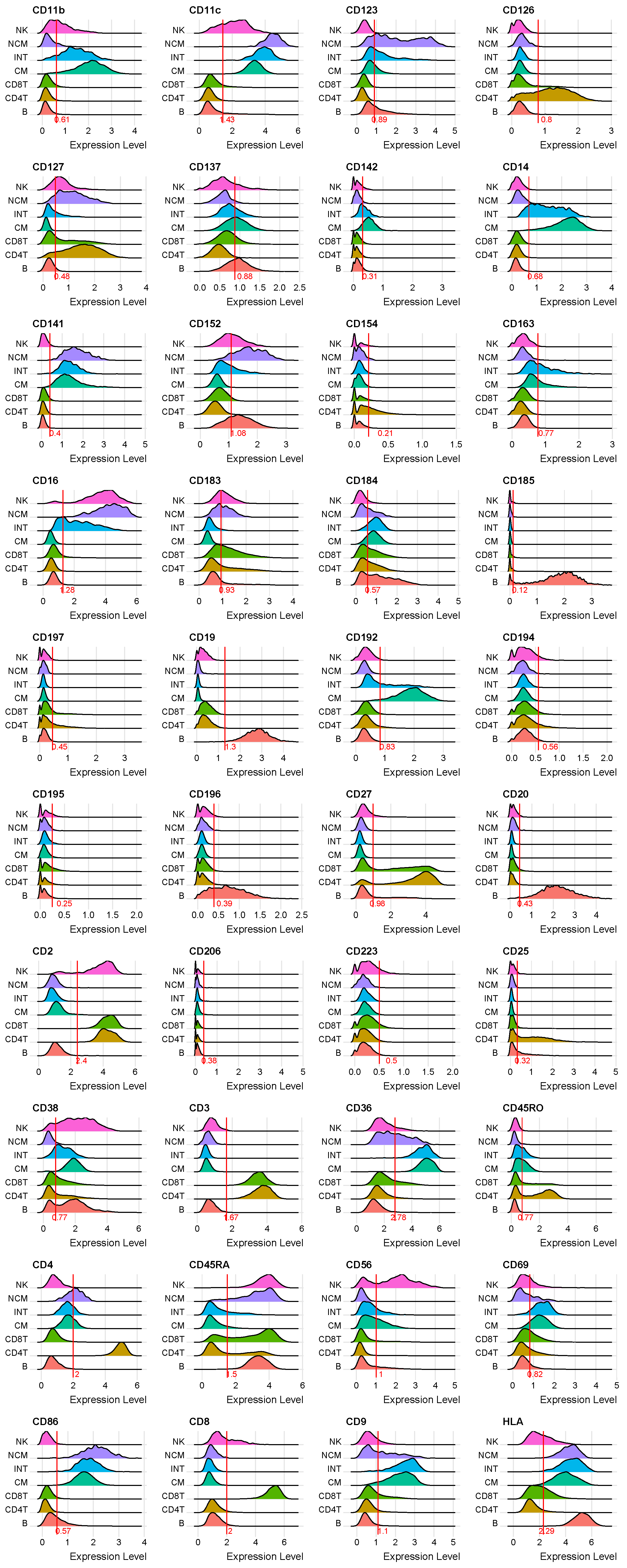


**Figure S1**

**
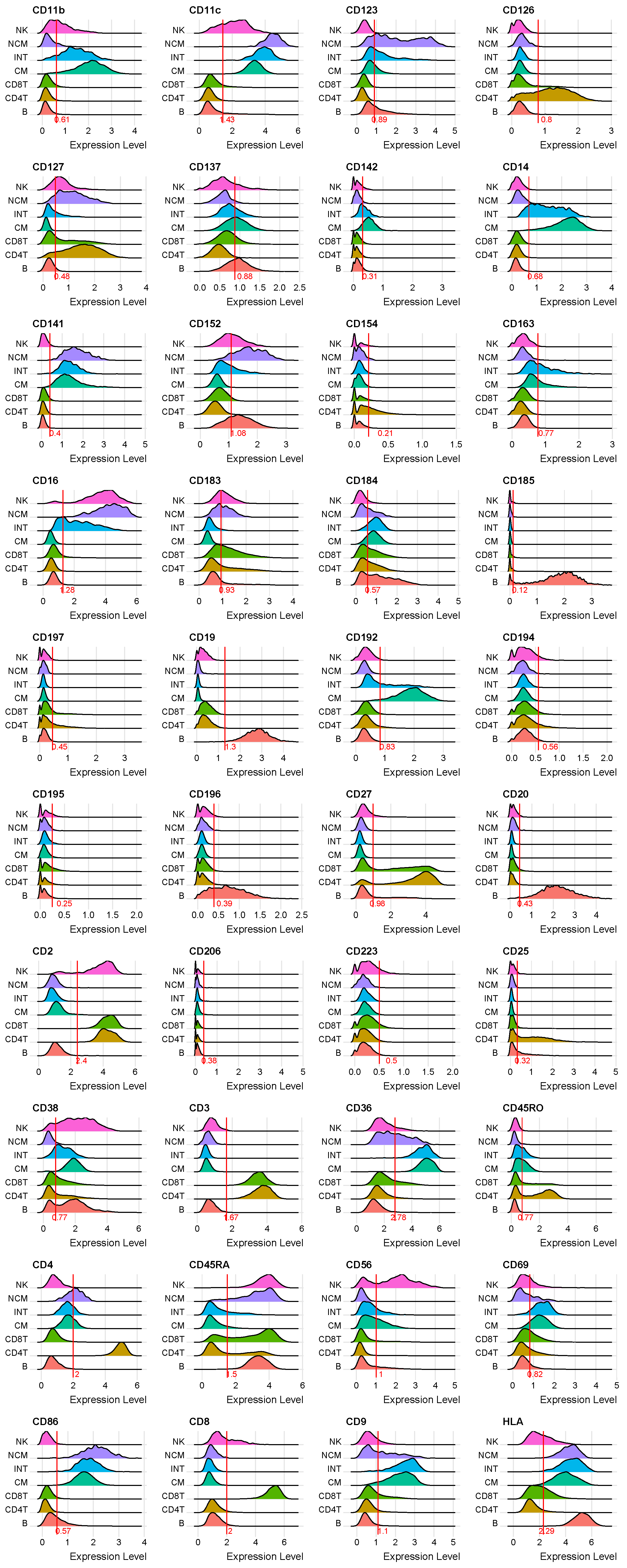
**

**Figure S1**

**Figure S2. (Related to Figure 1). (A) Ridgeline plots of the unthresholded expressions of all the 40 surface markers for each main cell types** [CD4+ T cells, CD8+ T cells, classical monocytes (CM), intermediate monocytes (INT), nonclassical monocytes (NCM), NK cells and B cells] using Seurat. These plots include the exact value used to threshold the antibodies. These plots separately show the distribution of each value of CLR normalized antibody derived tag for each main cell type. Expression levels are shown on x-axis. (**B) Age projection (below and above median) onto CM, INT, NCM, CD4+ T cells and CD8+ T cell UMAPs.**

Fig. S3.

Fig. S3. (Related to Figure 5). Dotplots of DEG for disease types for subsets. Dotplots of differentially expressed genes between HIV+CVD-CRT- vs HIV-CVD-CRT-, between HIV+CVD+CRT- vs HIV+CVD-CRT-, and between HIV+CVD+CRT+ vs HIV+CVD+CRT- in clusters of the main cell types. The thresholds set for the plots were adjusted p-value <0.05, avg.Log2FC>0 or <0, and pct.1 > 0.2. The size of dots represents log(pct.1/pct.2), where pct.1 is the proportion of cells expressing each gene in DM or CAD+ and pct.2 is the proportion of cells expressing each gene.

Table S1.

| **Reagent** | **Vendor** | **Catalogue #** |
| --- | --- | --- |
| *Antibodies* |  |  |
| AbSeq oligonucleotide-tagged antibodies | BD Biosciences | See Table S3 |
| *Chemicals or others* |  |  |
| 70% Ethanol | Milipore Sigma | E7023-500ml |
| cRPMI*1 | Major supplier | n/a |
| Fetal bovine serum (FBS) | Gemini | 100106-500mL |
| PBS | Fisher Scientific | 10010049 |
| L-Glutamine | Major supplier | n/a |
| Trypan Blue solution (0.4%) | Thermo Fisher | 15250061 |
| Fc Block | BD Biosciences | 564220 |
| DRAQ7 | BD Biosciences | 564904 |
| Calcein AM*2 | Thermo Fisher | C1430 |
| DMSO | Thermo Fisher | D12345 |
| Nuclease Free Water | Qiagen | 129114 |
| *Critical commercial assays* |  |  |
| BD Rhapsody Cartridge Reagent Kit | BD Biosciences | 633731 |
| BD Rhapsody Cartridge Kit | BD Biosciences | 633733 |
| BD Single Cell Multiplexing Kit- Human Sample Tag 12 | BD Biosciences | 633781 |
| BD Rhapsody targeted mRNA and AbSeq Amplification kit | BD Biosciences | 633771 |
| BD Rhapsody Human Immune Response Panel | BD Biosciences | 633750 |
| BD Rhapsody Custom Reagent Panel | BD Biosciences | 633742 |
| AMPure XP beads | Beckman Coulter | A63881 |
| D1000 ScreenTape | Agilent | 5067-5584 |
| D1000 Sample Buffer | Agilent | 5067-5603 |
| Qubit dsDNA High sensitivity and broad range Assay Kit | Thermo Fisher | Q33231 |
| NovaSeq S1 100 Cycle Kit | Illumina | 20012865 |
| NovaSeq S2 100 Cycle Kit | Illumina | 20012862 |

**Table S1. Reagents.** *1: From 500mL of RPMI-1640 (with L-Glutamine) remove 75.5 mL and transfer to two 50 mL conical tubes for future potential use. Add 50 mL of 100% human serum albumin (HSA). Add 5 mL of the following (concentrations indicated are of stock solutions): HEPES (1M), sodium pyruvate (100X) MEM-NEAA (100X), penicillin-streptomycin, and GlutaMAX. Add 0.5 mL of mercaptoethanol (1000X). All pursached by major suppliers. Mix by inversion in RPMI-1640 original bottle. Carefully transfer solution to a 500 mL CorningStore at 4° C. Vacuum filter system with a 0.45 mm filter size. *2: Resuspended in DMSO.

Table S2.

| Day 1 | Viability (%) | Day 2 | Viability (%) |
| --- | --- | --- | --- |
| 1 | 94 | 1 | 91 |
| 2 | 93 | 2 | 93 |
| 3 | 81 | 3 | 85 |
| 4 | 87 | 4 | 91 |
| 5 | 81 | 5 | 92 |
| 6 | 87 | 6 | 88 |
| 7 | 89 | 7 | 83 |
| 8 | 89 | 8 | 84 |
| 9 | 95 | 9 | 94 |
| 10 | 94 | 10 | 91 |
| 11 | 75 | 11 | 86 |
| 12 | 89 | 12 | 89 |
| 13 | 79 | 13 | 89 |
| 14 | 92 | 14 | 94 |
| 15 | 85 | 15 | 87 |
| 16 | 75 | 16 | 93 |
| Min | **75** | **Min** | **83** |
| Max | **95** | **Max** | **94** |
| Median | **88** | **Median** | **90** |
| Average | **87** | **Average** | **89** |

**Table S2: The cell viability of each frozen PBMC tube.** On two separate days 16 tubes of frozen PBMCs were thawed and used. The viability was determined using the BD Rhapsody Scanner. Minimum (Min), maximum (Max), median, and average viability were calculated in each day.

Table S3.

| **Specificity** | **Clone** | **Catalogue Number** |
| --- | --- | --- |
| CD11b | M1/70 | 940008 |
| CD11c | B-LY6 | 940024 |
| CD123 (IL-3RA) | 7G3 | 940020 |
| CD126 (IL-6R) | M5 | 940090 |
| CD127 (IL-7R) | HIL-7R-M21 | 940012 |
| CD137 | 4B4-1 | 940055 |
| CD14 | MPHIP9 | 940005 |
| CD141 | 1A4 | 940079 |
| CD142 | HTF-1 | 940280 |
| CD152 (CTLA-4) | BNI3 | 940034 |
| CD154 | TRAP1 | 940053 |
| CD16 | 3G8 | 940006 |
| CD163 | GHI/61 | 940058 |
| CD183 (CXCR3) | 1C6/CXCR3 | 940030 |
| CD184 (CXCR4) | 12G5 | 940056 |
| CD185 (CXCR5) | RF8B2 | 940042 |
| CD19 | SJ25C1 | 940004 |
| CD192 (CCR2) | 1D9 | 940286 |
| CD194 (CCR4) | 1G1 | 940047 |
| CD195 (CCR5) | 2D7/CCR5 | 940050 |
| CD196 (CCR6) | 11A9 | 940033 |
| CD197 (CCR7) | 3D12 | 940014 |
| CD2 | RPA-2.10 | 940046 |
| CD20 | 2H7 | 940016 |
| CD206 | 19.2 | 940068 |
| CD223 (LAG-3) | T47-530 | 940080 |
| CD25 | 2A3 | 940009 |
| CD27 | M-T271 | 940018 |
| CD3 | SK7 | 940000 |
| CD36 | CB38 (NL07) | 940224 |
| CD38 | HIT2 | 940013 |
| CD4 | SK3 | 940001 |
| CD45RA | HI100 | 940011 |
| CD45RO | UCHL1 | 940022 |
| CD56 | NCAM16.2 | 940007 |
| CD69 | FN50 | 940019 |
| CD8 | RPA-T8 | 940003 |
| CD86 | 2331(FUN-1) | 940025 |
| CD9 | M-L13 | 940078 |
| HLA-DR (CD74) | G46-6 | 940010 |

**Table S3. List of 40 titrated oligonucleotide-tagged monoclonal antibodies.** All the monoclonal antibodies were from BD Biosciences (NJ, USA). A cocktail of these 40 antibodies was used for staining of the cells following manufacturer’s recommendations.

Table S4.

| **Genes** |
| --- |
| ACSL1 |
| AL137655 |
| APLP2 |
| APOBEC3A |
| ASAH1 |
| B3GALT2 |
| BC013828 |
| C3AR1 |
| C5AR1 |
| CAMKK2 |
| CAVIN2 |
| CD300C |
| CD43 |
| CD83 |
| CD96 |
| CSF3R |
| CTSA |
| CYTIP |
| EMR1 |
| FBP1 |
| FCGR2A |
| FCGR3B |
| FGFBP2 |
| FPR1 |
| G0S2 |
| GCA |
| GLUL |
| GNG11 |
| GPR56 |
| HOPX |
| IFITM1 |
| IKZF3 |
| ITGA2B |
| ITGB3 |
| ITM2B |
| JMJD1C-AS1 |
| KLF2 |
| KLRC4-KLRK1 |
| KLRD1 |
| LILRB1 |
| LRP1 |
| LYZ |
| MCTP2 |
| MEG8 |
| MIR4718 |
| MIR5192 |
| MMP25 |
| MMRN1 |
| MNDA |
| MXD1 |
| NAIP |
| NCF1 |
| NCF1C |
| NEAT1 |
| NLRP3 |
| NR4A1 |
| PELI1 |
| PLIN2 |
| PTAFR |
| PXN |
| R3HDM4 |
| RAB27B |
| RASGEF1B |
| S100A8 |
| S1PR5 |
| SAMD3 |
| SCPEP1 |
| SDCBP |
| SH2D1B |
| SH3BGRL2 |
| SIGLEC10 |
| SLC11A1 |
| SLC2A3 |
| SLC35G2 |
| SOD2 |
| SORL1 |
| SPON2 |
| SRGN |
| SYNE1 |
| SYNE2 |
| TC2N |
| TCRBV3S1 |
| TKT |
| TNFRSF10C |
| TRGC2 |
| TSPAN14 |
| TTC38 |
| TTYH3 |
| VCAN |
| XPO6 |
| XYLT1 |

**Table S4: List of selected genes included in the custom panel.** A total of 91 genes were included in the customized panel in addition to the already described BD Human Immune Response Panel.

Table S5.

| **Antibodies** | **Threshold** |
| --- | --- |
| CD11b | 0.6 |
| CD11c | 1.43 |
| CD123 (IL-3RA) | 0.89 |
| CD126 | 0.8 |
| CD127 (IL-7R) | 0.49 |
| CD137 | 0.91 |
| CD14 | 0.68 |
| CD141 | 0.4 |
| CD142 | 0.31 |
| CD152 (CTLA-4) | 1.08 |
| CD154 | 0.21 |
| CD16 | 1.28 |
| CD163 | 0.77 |
| CD183 (CXCR3) | 0.93 |
| CD184 (CXCR4) | 0.57 |
| CD185 (CXCR5) | 0.12 |
| CD19 | 1.3 |
| CD192 (CCR2) | 0.83 |
| CD194 (CCR4) | 0.56 |
| CD195 (CCR5) | 0.25 |
| CD196 (CCR6) | 0.39 |
| CD197 (CCR7) | Not included in the analysis |
| CD2 | 2.41 |
| CD20 | 0.43 |
| CD206 | 0.38 |
| CD223 (LAG-3) | 0.49 |
| CD25 | 0.32 |
| CD27 | 0.98 |
| CD3 | 1.67 |
| CD36 | 2.78 |
| CD38 | 0.77 |
| CD4 | 2 |
| CD45RA | 1.5 |
| CD45RO | 0.77 |
| CD56 | 1 |
| CD69 | 0.82 |
| CD8 | 2 |
| CD86 | 0.57 |
| CD9 | 1.1 |
| CD74 (HLA-DR) | 2.29 |

**Table S5. Threshold values of each antibody.** For the antibodies we use CLR Normalization, which is the ratio of the UMI value to the geometric mean of all the UMIs in that cell in log scale. The thresholds are in the same space.

Table S6.

| **B** | **CD4+ T**  **cells** | **CD8+ T**  **cells** | **Classical**  **Monocytes** | **Intermediate Monocytes** | **Nonclassical Monocytes** | **NK cells** |
| --- | --- | --- | --- | --- | --- | --- |
| CD3 | CD8  CD19 | CD4  CD19 | CD3  CD16 | CD3  CD19 | CD3  CD14 | CD3  CD4 |
|  |  |  | CD19 |  | CD19 | CD14 |
|  |  |  |  |  | CD56 | CD19  CD20 |
|  |  |  |  |  |  | CD123 (IL-3RA) |
|  |  |  |  |  |  | CD126 (IL-6R) |

**Table S6. Antibodies not used for cell clustering.** B; B cells (CD19+CD3-), CD4+ T; CD4+ T cells (CD19-CD3+CD4+CD8-), CD8+ T; CD8+ T cells (CD19-CD3+CD4-CD8+), CM; classical monocytes (CD3-CD19-CD14+CD16-), INT; intermediate monocytes (CD3-CD19-CD14+CD16+), NCM; nonclassical monocytes (CD3-CD19-CD56-CD16+), NK; NK cells (CD56+CD14-CD20-CD123-CD206-).

Table S7.

|  | **B cells** | **CD4+ T cells** | **CD8+ T cells** | **Classical Monocytes** | **Intermediate Monocytes** | **Nonclassical Monocytes** | **NK cells** |
| --- | --- | --- | --- | --- | --- | --- | --- |
| **Cluster 1** | 641 | 1,750 | 900 | 1,512 | 418 | 230 | 852 |
| **Cluster 2** | 510 | 1,668 | 1,350 | 1,129 | 326 | 148 | 777 |
| **Cluster 3** | 403 | 1,238 | 1,647 | 779 | 251 | 97 | 172 |
| **Cluster 4** | 391 | 1,235 | 1,182 | 636 |  |  |  |
| **Cluster 5** | 360 | 1,228 | 1,060 | 552 |  |  |  |
| **Cluster 6** | 312 | 1,007 | 887 | 477 |  |  |  |
| **Cluster 7** | 218 | 685 | 841 | 60 |  |  |  |
| **Cluster 8** |  | 576 | 700 |  |  |  |  |
| **Cluster 9** |  | 549 | 645 |  |  |  |  |
| **Cluster 10** |  | 477 | 446 |  |  |  |  |
| **Cluster 11** |  | 273 | 525 |  |  |  |  |
| **Cluster 12** |  | 228 | 347 |  |  |  |  |
| **Cluster 13**  **Cluster 14** |  | 105 | 199  136 |  |  |  |  |
| **Total** | 2,835 | 11,019 | 10,865 | 5,145 | 995 | 475 | 1,801 |

**Table S7: Number of Cells in each of the clusters.** Number of cells for each of the major cell types in our dataset. It further gives the number of cells present in each cluster of the cell type.

Table S8.

**A) CD4+ T cells**

| **Gene** | **p_val** | **avg_log2FC** | **pct.1** | **pct.2** | **p_val_adj** | **cluster** |
| --- | --- | --- | --- | --- | --- | --- |
| **CHI3L2** | 9.32E-54 | 1.9480876 | 0.092 | 0.021 | 4.19E-51 | 1 |
| **TXK** | 8.48E-44 | 1.4169331 | 0.153 | 0.059 | 3.81E-41 | 1 |
| **BACH2** | 5.46E-19 | 1.2901592 | 0.053 | 0.018 | 2.45E-16 | 1 |
| **CD200** | 3.18E-06 | 0.8097612 | 0.009 | 0.002 | 0.0014264 | 1 |
| **EGR3** | 3.16E-05 | 0.5447403 | 0.011 | 0.004 | 0.0141734 | 1 |
| **GZMK** | 5.43E-38 | 1.4027497 | 0.103 | 0.033 | 2.44E-35 | 2 |
| **TNFSF13B** | 7.82E-10 | 0.8176179 | 0.021 | 0.006 | 3.51E-07 | 2 |
| **NT5E** | 1.33E-06 | 0.6994758 | 0.014 | 0.004 | 0.0005986 | 2 |
| **CCR6** | 1.27E-21 | 1.1175686 | 0.07 | 0.023 | 5.68E-19 | 3 |
| **PTGDR2** | 4.44E-08 | 0.8396679 | 0.01 | 0.002 | 1.99E-05 | 3 |
| **RORC** | 1.10E-05 | 0.6040914 | 0.017 | 0.006 | 0.0049195 | 3 |
| **CSF2** | 5.35E-06 | 0.5125288 | 0.005 | 0.001 | 0.0024021 | 3 |
| **GZMH** | 0 | 3.9577701 | 0.381 | 0.025 | 0 | 4 |
| **FGFBP2** | 0 | 3.862154 | 0.379 | 0.026 | 0 | 4 |
| **NKG7** | 0 | 3.6504907 | 0.635 | 0.084 | 0 | 4 |
| **GNLY** | 0 | 3.289339 | 0.547 | 0.075 | 0 | 4 |
| **CCL5** | 0 | 3.0708377 | 0.781 | 0.184 | 0 | 4 |
| **CCL4** | 8.71E-122 | 2.9101095 | 0.138 | 0.017 | 3.91E-119 | 4 |
| **IFNG** | 3.22E-150 | 2.7251475 | 0.194 | 0.028 | 1.45E-147 | 4 |
| **GZMB** | 4.65E-159 | 2.6888499 | 0.179 | 0.021 | 2.09E-156 | 4 |
| **S1PR5** | 6.35E-102 | 2.6055742 | 0.087 | 0.007 | 2.85E-99 | 4 |
| **PRF1** | 1.99E-126 | 2.5253246 | 0.144 | 0.018 | 8.91E-124 | 4 |
| **CST7** | 0 | 2.4968295 | 0.567 | 0.123 | 0 | 4 |
| **ZNF683** | 1.43E-69 | 2.4257794 | 0.064 | 0.006 | 6.42E-67 | 4 |
| **GZMA** | 8.84E-212 | 2.3965877 | 0.339 | 0.065 | 3.97E-209 | 4 |
| **IL18RAP** | 4.99E-56 | 2.2298619 | 0.066 | 0.008 | 2.24E-53 | 4 |
| **ITGAM** | 4.04E-71 | 2.048095 | 0.097 | 0.015 | 1.81E-68 | 4 |
| **TBX21** | 1.18E-90 | 2.0239943 | 0.133 | 0.022 | 5.32E-88 | 4 |
| **TRGC2** | 1.19E-81 | 1.9536107 | 0.193 | 0.05 | 5.35E-79 | 4 |
| **HOPX** | 8.73E-123 | 1.918998 | 0.254 | 0.059 | 3.92E-120 | 4 |
| **TARP-refseq** | 4.94E-77 | 1.9090284 | 0.181 | 0.046 | 2.22E-74 | 4 |
| **LAIR2** | 9.97E-39 | 1.8897573 | 0.064 | 0.012 | 4.48E-36 | 4 |
| **YBX3** | 1.99E-23 | 1.2983265 | 0.094 | 0.035 | 8.94E-21 | 5 |
| **CCR4** | 3.47E-16 | 1.2543184 | 0.036 | 0.008 | 1.56E-13 | 6 |
| **GAB2** | 1.56E-07 | 0.8328366 | 0.035 | 0.013 | 6.99E-05 | 6 |
| **PTGDR2** | 1.18E-08 | 0.7674667 | 0.011 | 0.002 | 5.30E-06 | 6 |
| **F5** | 7.68E-07 | 0.7365653 | 0.024 | 0.008 | 0.0003448 | 6 |
| **TNFRSF4** | 2.73E-05 | 0.7045142 | 0.023 | 0.009 | 0.0122635 | 6 |
| **IL22** | 3.34E-05 | 0.6280634 | 0.006 | 0.001 | 0.0149742 | 6 |
| **CXCR3** | 2.47E-48 | 1.9719843 | 0.15 | 0.035 | 1.11E-45 | 7 |
| **CXCR5** | 1.21E-17 | 1.6567556 | 0.063 | 0.016 | 5.43E-15 | 7 |
| **DUSP4** | 1.28E-10 | 1.3046945 | 0.07 | 0.027 | 5.77E-08 | 7 |
| **RASGEF1B** | 4.85E-08 | 1.2946727 | 0.053 | 0.021 | 2.18E-05 | 7 |
| **TNFRSF4** | 7.45E-05 | 1.0139635 | 0.025 | 0.009 | 0.0334474 | 7 |
| **ITGA2B** | 3.46E-05 | 0.5725227 | 0.006 | 0.001 | 0.0155145 | 7 |
| **VSIG4** | 1.74E-06 | 0.6016332 | 0.003 | 0 | 0.0007803 | 8 |
| **CXCR5** | 2.84E-93 | 2.7952262 | 0.137 | 0.013 | 1.27E-90 | 9 |
| **CLEC4D** | 3.50E-05 | 0.4406638 | 0.004 | 0 | 0.0157313 | 9 |
| **CSF3** | 3.51E-05 | 0.3961713 | 0.004 | 0 | 0.0157599 | 9 |
| **BPI** | 1.26E-05 | 0.2414123 | 0.002 | 0 | 0.0056656 | 9 |
| **FOXP3** | 0 | 4.048715 | 0.256 | 0.009 | 0 | 10 |
| **TIGIT** | 4.81E-27 | 2.1931992 | 0.078 | 0.014 | 2.16E-24 | 10 |
| **CTLA4** | 1.64E-54 | 2.1313589 | 0.249 | 0.064 | 7.38E-52 | 10 |
| **CD70** | 7.84E-20 | 2.1081908 | 0.029 | 0.003 | 3.52E-17 | 10 |
| **CCR8** | 2.70E-20 | 1.9894505 | 0.019 | 0.001 | 1.21E-17 | 10 |
| **LGALS3** | 5.09E-39 | 1.9287846 | 0.214 | 0.061 | 2.29E-36 | 10 |
| **IL2RA** | 1.26E-15 | 1.9169749 | 0.061 | 0.014 | 5.65E-13 | 10 |
| **KIAA0101** | 3.67E-15 | 1.7957877 | 0.025 | 0.003 | 1.65E-12 | 10 |
| **DUSP4** | 1.51E-14 | 1.7044469 | 0.088 | 0.027 | 6.77E-12 | 10 |
| **IKZF2** | 5.14E-15 | 1.6339378 | 0.021 | 0.002 | 2.31E-12 | 10 |
| **ENTPD1** | 2.76E-18 | 1.5723121 | 0.023 | 0.002 | 1.24E-15 | 10 |
| **LAIR2** | 3.64E-13 | 1.4122406 | 0.061 | 0.016 | 1.63E-10 | 10 |
| **F5** | 3.52E-07 | 1.3647756 | 0.031 | 0.008 | 0.0001581 | 10 |
| **NCR3** | 1.42E-05 | 1.2571621 | 0.046 | 0.018 | 0.0063535 | 10 |
| **LRRC32** | 5.65E-16 | 1.2499924 | 0.017 | 0.001 | 2.54E-13 | 10 |
| **IL12RB2** | 2.52E-06 | 1.0548627 | 0.013 | 0.002 | 0.0011302 | 10 |
| **UBE2C** | 8.15E-06 | 1.0426959 | 0.008 | 0.001 | 0.0036602 | 10 |
| **NINJ2** | 4.42E-05 | 1.0132232 | 0.034 | 0.012 | 0.0198376 | 10 |
| **CCR4** | 4.48E-05 | 0.9787005 | 0.029 | 0.01 | 0.0201158 | 10 |
| **CXCR6** | 5.48E-06 | 0.6903814 | 0.01 | 0.001 | 0.002461 | 10 |
| **GZMB** | 8.79E-269 | 3.843435 | 0.443 | 0.029 | 3.95E-266 | 11 |
| **GNLY** | 0 | 3.8054976 | 0.897 | 0.109 | 0 | 11 |
| **NKG7** | 1.86E-300 | 3.368251 | 0.886 | 0.127 | 8.36E-298 | 11 |
| **FGFBP2** | 2.19E-274 | 3.2664298 | 0.593 | 0.053 | 9.83E-272 | 11 |
| **GZMH** | 8.01E-208 | 2.9886714 | 0.52 | 0.053 | 3.60E-205 | 11 |
| **FCGR3A** | 9.93E-45 | 2.8828508 | 0.136 | 0.017 | 4.46E-42 | 11 |
| **ITGAM** | 5.53E-62 | 2.8583471 | 0.176 | 0.02 | 2.48E-59 | 11 |
| **PRF1** | 4.64E-83 | 2.7987184 | 0.234 | 0.027 | 2.08E-80 | 11 |
| **KLRF1** | 8.67E-76 | 2.7624178 | 0.128 | 0.008 | 3.89E-73 | 11 |
| **B3GAT1** | 8.21E-34 | 2.7443813 | 0.062 | 0.005 | 3.69E-31 | 11 |
| **KLRK1** | 4.97E-83 | 2.7080011 | 0.187 | 0.017 | 2.23E-80 | 11 |
| **KLRC4** | 3.18E-85 | 2.6918309 | 0.19 | 0.017 | 1.43E-82 | 11 |
| **CCL5** | 1.84E-189 | 2.664455 | 0.927 | 0.234 | 8.27E-187 | 11 |
| **ZNF683** | 6.22E-54 | 2.5444924 | 0.114 | 0.01 | 2.79E-51 | 11 |
| **S1PR5** | 1.09E-68 | 2.5394077 | 0.147 | 0.012 | 4.91E-66 | 11 |
| **CCL4** | 3.20E-50 | 2.5055125 | 0.183 | 0.026 | 1.44E-47 | 11 |
| **CTSW** | 1.41E-79 | 2.3679307 | 0.432 | 0.089 | 6.33E-77 | 11 |
| **HOPX** | 3.00E-92 | 2.2868424 | 0.418 | 0.073 | 1.35E-89 | 11 |
| **KLRD1** | 8.95E-28 | 2.2493962 | 0.055 | 0.004 | 4.02E-25 | 11 |
| **CST7** | 4.65E-126 | 2.208149 | 0.707 | 0.159 | 2.09E-123 | 11 |
| **CLC** | 1.01E-07 | 1.1883671 | 0.013 | 0.001 | 4.55E-05 | 12 |
| **KLRC1** | 3.31E-06 | 1.7839959 | 0.029 | 0.003 | 0.0014844 | 13 |
| **CXCL10** | 1.15E-05 | 1.0918302 | 0.01 | 0 | 0.005183 | 13 |

**B) CD8+ T cells**

| **Gene** | **p_val** | **avg_log2FC** | **pct.1** | **pct.2** | **p_val_adj** | **cluster** |
| --- | --- | --- | --- | --- | --- | --- |
| **LEF1** | 1.31E-235 | 2.258178856 | 0.539 | 0.129 | 5.88E-233 | 1 |
| **CCR7** | 7.83E-143 | 2.085576378 | 0.354 | 0.084 | 3.52E-140 | 1 |
| **TXK** | 7.85E-65 | 2.071559258 | 0.149 | 0.032 | 3.53E-62 | 1 |
| **NT5E** | 7.03E-42 | 2.052427872 | 0.082 | 0.015 | 3.16E-39 | 1 |
| **MYC** | 4.30E-107 | 1.930993076 | 0.368 | 0.114 | 1.93E-104 | 1 |
| **PASK** | 4.16E-96 | 1.808461489 | 0.341 | 0.106 | 1.87E-93 | 1 |
| **CD79A** | 8.11E-26 | 1.707031975 | 0.059 | 0.013 | 3.64E-23 | 1 |
| **BACH2** | 1.28E-21 | 1.681548849 | 0.043 | 0.008 | 5.74E-19 | 1 |
| **SELL** | 9.20E-78 | 1.640111924 | 0.374 | 0.145 | 4.13E-75 | 1 |
| **CD27** | 5.88E-48 | 1.552252879 | 0.26 | 0.102 | 2.64E-45 | 1 |
| **FCER1G** | 4.57E-14 | 1.278720617 | 0.077 | 0.03 | 2.05E-11 | 1 |
| **CXCL16** | 2.05E-10 | 1.259520597 | 0.052 | 0.02 | 9.19E-08 | 1 |
| **LTB** | 2.15E-09 | 1.242567114 | 0.043 | 0.016 | 9.64E-07 | 1 |
| **DPP4** | 6.22E-13 | 1.223636981 | 0.046 | 0.014 | 2.79E-10 | 1 |
| **BTLA** | 1.02E-06 | 1.056889969 | 0.019 | 0.005 | 0.0004594 | 1 |
| **FBP1** | 1.54E-05 | 1.000654475 | 0.016 | 0.005 | 0.0069343 | 1 |
| **SH3BGRL2** | 1.08E-16 | 0.978141891 | 0.017 | 0.002 | 4.85E-14 | 1 |
| **TNFRSF13C** | 8.52E-09 | 0.914281849 | 0.011 | 0.002 | 3.83E-06 | 1 |
| **CR2** | 2.41E-06 | 0.810442947 | 0.008 | 0.001 | 0.0010841 | 1 |
| **LEF1** | 1.33E-286 | 2.33877049 | 0.492 | 0.116 | 5.99E-284 | 2 |
| **CCR7** | 9.68E-158 | 2.124350855 | 0.314 | 0.077 | 4.35E-155 | 2 |
| **PASK** | 3.01E-134 | 1.997834395 | 0.33 | 0.097 | 1.35E-131 | 2 |
| **TXK** | 5.22E-62 | 1.927313172 | 0.125 | 0.029 | 2.34E-59 | 2 |
| **SELL** | 3.54E-158 | 1.859822359 | 0.407 | 0.129 | 1.59E-155 | 2 |
| **NT5E** | 4.55E-38 | 1.774856122 | 0.067 | 0.014 | 2.04E-35 | 2 |
| **CD27** | 3.74E-85 | 1.687908631 | 0.269 | 0.094 | 1.68E-82 | 2 |
| **MYC** | 1.50E-109 | 1.684183715 | 0.326 | 0.108 | 6.72E-107 | 2 |
| **CD79A** | 4.64E-19 | 1.469663607 | 0.045 | 0.012 | 2.08E-16 | 2 |
| **LTB** | 3.07E-14 | 1.385708458 | 0.044 | 0.014 | 1.38E-11 | 2 |
| **FCER1G** | 1.57E-16 | 1.322630638 | 0.071 | 0.028 | 7.05E-14 | 2 |
| **BACH2** | 1.66E-16 | 1.32150018 | 0.033 | 0.008 | 7.44E-14 | 2 |
| **TNFRSF25** | 1.11E-09 | 1.214628039 | 0.033 | 0.012 | 4.98E-07 | 2 |
| **CD79B** | 5.62E-06 | 0.915030969 | 0.016 | 0.005 | 0.0025249 | 2 |
| **FBP1** | 5.68E-10 | 0.900857242 | 0.017 | 0.004 | 2.55E-07 | 2 |
| **CX3CR1** | 1.33E-26 | 1.256609722 | 0.057 | 0.015 | 5.97E-24 | 3 |
| **CD160** | 3.55E-27 | 1.107908831 | 0.098 | 0.037 | 1.59E-24 | 3 |
| **B3GAT1** | 1.25E-11 | 0.900417682 | 0.043 | 0.017 | 5.61E-09 | 3 |
| **TYMS** | 4.00E-13 | 1.063566447 | 0.012 | 0.001 | 1.79E-10 | 4 |
| **NINJ2** | 9.05E-10 | 0.973834574 | 0.033 | 0.011 | 4.06E-07 | 4 |
| **CD70** | 6.39E-12 | 0.883225596 | 0.021 | 0.004 | 2.87E-09 | 4 |
| **CTLA4** | 7.88E-11 | 0.806782809 | 0.043 | 0.016 | 3.54E-08 | 4 |
| **KIAA0101** | 4.35E-05 | 0.723711109 | 0.008 | 0.002 | 0.0195192 | 4 |
| **ENTPD1** | 1.65E-06 | 0.364803306 | 0.007 | 0.001 | 0.0007395 | 4 |
| **GZMK** | 4.72E-105 | 1.752966702 | 0.36 | 0.118 | 2.12E-102 | 5 |
| **CXCR3** | 2.79E-38 | 1.564205439 | 0.142 | 0.046 | 1.25E-35 | 5 |
| **DUSP4** | 7.45E-13 | 1.22414258 | 0.051 | 0.018 | 3.35E-10 | 5 |
| **CCR6** | 1.17E-09 | 1.103267627 | 0.011 | 0.002 | 5.27E-07 | 5 |
| **CXCR5** | 4.92E-06 | 0.632501051 | 0.01 | 0.002 | 0.0022071 | 5 |
| **B3GALT2** | 1.70E-05 | 0.130399077 | 0.002 | 0 | 0.0076294 | 5 |
| **GNLY** | 0 | 2.314736097 | 0.918 | 0.347 | 0 | 6 |
| **KLRF1** | 9.31E-80 | 2.16833054 | 0.157 | 0.029 | 4.18E-77 | 6 |
| **TRDC** | 4.09E-49 | 2.045916848 | 0.103 | 0.02 | 1.83E-46 | 6 |
| **KLRC1** | 1.13E-47 | 1.662239204 | 0.108 | 0.022 | 5.08E-45 | 6 |
| **GZMB** | 7.82E-124 | 1.653798318 | 0.478 | 0.159 | 3.51E-121 | 6 |
| **KLRC3** | 1.02E-58 | 1.609003713 | 0.159 | 0.038 | 4.58E-56 | 6 |
| **FCGR3A** | 5.83E-60 | 1.546128145 | 0.26 | 0.087 | 2.62E-57 | 6 |
| **KIR2DL1** | 4.45E-24 | 1.414133032 | 0.052 | 0.01 | 2.00E-21 | 6 |
| **CD300A** | 1.89E-22 | 1.26803823 | 0.089 | 0.028 | 8.49E-20 | 6 |
| **NCR3** | 2.32E-26 | 1.204427127 | 0.135 | 0.049 | 1.04E-23 | 6 |
| **LYN** | 1.15E-21 | 1.088364325 | 0.096 | 0.032 | 5.18E-19 | 6 |
| **SH2D1B** | 1.83E-15 | 0.948236749 | 0.014 | 0.001 | 8.21E-13 | 6 |
| **ITGAM** | 2.61E-21 | 0.901183101 | 0.117 | 0.044 | 1.17E-18 | 6 |
| **FCGR2A** | 1.39E-07 | 0.688910991 | 0.024 | 0.007 | 6.23E-05 | 6 |
| **IKZF2** | 1.71E-05 | 0.677635859 | 0.018 | 0.006 | 0.0076816 | 6 |
| **FASLG** | 6.04E-05 | 0.52625033 | 0.016 | 0.005 | 0.027135 | 6 |
| **LAT2** | 1.13E-05 | 0.492263111 | 0.017 | 0.005 | 0.0050897 | 6 |
| **CMKLR1** | 1.89E-06 | 0.464558812 | 0.008 | 0.001 | 0.0008488 | 6 |
| **CCR9** | 3.84E-11 | 0.833425216 | 0.01 | 0.001 | 1.72E-08 | 7 |
| **VPREB3** | 1.05E-06 | 0.450639417 | 0.002 | 0 | 0.0004705 | 7 |
| **CCR10** | 8.66E-05 | 0.91633497 | 0.004 | 0 | 0.0388751 | 8 |
| **KIR2DL1** | 8.79E-07 | 0.991844119 | 0.036 | 0.012 | 0.0003946 | 9 |
| **TTC38** | 3.25E-12 | 0.936295135 | 0.093 | 0.037 | 1.46E-09 | 9 |
| **IKZF2** | 1.70E-05 | 0.631911506 | 0.02 | 0.006 | 0.0076301 | 9 |
| **BLK** | 6.89E-05 | 0.094111941 | 0.002 | 0 | 0.0309191 | 9 |
| **CCR5** | 3.44E-05 | 0.840102807 | 0.022 | 0.006 | 0.0154609 | 10 |
| **FAM129C** | 4.73E-08 | 0.42924999 | 0.004 | 0 | 2.12E-05 | 10 |
| **CTSG** | 5.23E-05 | 0.299329021 | 0.004 | 0 | 0.0234751 | 10 |
| **PTGDR2** | 1.16E-139 | 2.65916911 | 0.069 | 0 | 5.23E-137 | 11 |
| **CCR4** | 2.47E-63 | 2.081402347 | 0.038 | 0.001 | 1.11E-60 | 11 |
| **MYC** | 2.48E-77 | 1.979143675 | 0.4 | 0.122 | 1.11E-74 | 11 |
| **ADGRE1** | 2.35E-22 | 1.956361343 | 0.036 | 0.004 | 1.05E-19 | 11 |
| **GAB2** | 2.23E-35 | 1.890718218 | 0.067 | 0.008 | 1.00E-32 | 11 |
| **IL4R** | 7.28E-53 | 1.84068866 | 0.272 | 0.08 | 3.27E-50 | 11 |
| **LTB** | 2.34E-20 | 1.640495514 | 0.07 | 0.015 | 1.05E-17 | 11 |
| **ICOS** | 2.31E-30 | 1.634468266 | 0.156 | 0.045 | 1.04E-27 | 11 |
| **IL2** | 8.13E-22 | 1.600602421 | 0.017 | 0.001 | 3.65E-19 | 11 |
| **CD28** | 1.26E-30 | 1.599051986 | 0.122 | 0.029 | 5.65E-28 | 11 |
| **TNFSF10** | 1.16E-15 | 1.528784271 | 0.099 | 0.033 | 5.22E-13 | 11 |
| **IL2RA** | 2.63E-36 | 1.525024084 | 0.029 | 0.001 | 1.18E-33 | 11 |
| **BIRC3** | 4.61E-28 | 1.485552725 | 0.154 | 0.046 | 2.07E-25 | 11 |
| **LGALS9** | 6.21E-14 | 1.427241737 | 0.099 | 0.035 | 2.79E-11 | 11 |
| **SELL** | 1.96E-52 | 1.348033386 | 0.406 | 0.151 | 8.80E-50 | 11 |
| **TNFRSF25** | 3.28E-12 | 1.274936576 | 0.05 | 0.012 | 1.47E-09 | 11 |
| **FAS** | 2.73E-11 | 1.251987942 | 0.09 | 0.034 | 1.23E-08 | 11 |
| **CLC** | 1.55E-27 | 1.057986871 | 0.011 | 0 | 6.96E-25 | 11 |
| **SOD2** | 1.73E-06 | 1.051303638 | 0.05 | 0.019 | 0.0007769 | 11 |
| **ZBTB16** | 3.51E-09 | 0.999227636 | 0.013 | 0.001 | 1.58E-06 | 11 |
| **GZMK** | 6.86E-30 | 1.358726437 | 0.349 | 0.135 | 3.08E-27 | 12 |
| **DUSP4** | 2.37E-08 | 1.256705685 | 0.063 | 0.02 | 1.06E-05 | 12 |
| **TRDC** | 2.55E-22 | 2.473857692 | 0.136 | 0.024 | 1.15E-19 | 13 |
| **KLRF1** | 4.58E-15 | 1.650072936 | 0.146 | 0.037 | 2.06E-12 | 13 |
| **KLRB1** | 5.80E-25 | 1.508026959 | 0.387 | 0.133 | 2.60E-22 | 13 |
| **KLRC3** | 7.71E-13 | 1.470386248 | 0.156 | 0.045 | 3.46E-10 | 13 |
| **FCGR3A** | 1.00E-14 | 1.375346966 | 0.266 | 0.098 | 4.50E-12 | 13 |
| **PIK3AP1** | 3.05E-14 | 1.362887864 | 0.211 | 0.069 | 1.37E-11 | 13 |
| **S1PR5** | 4.31E-21 | 1.347226629 | 0.307 | 0.098 | 1.94E-18 | 13 |
| **LYN** | 3.47E-10 | 1.269792592 | 0.121 | 0.035 | 1.56E-07 | 13 |
| **ITGAX** | 5.17E-07 | 1.225929924 | 0.095 | 0.031 | 0.0002323 | 13 |
| **CD38** | 2.65E-08 | 1.144188445 | 0.035 | 0.005 | 1.19E-05 | 13 |
| **KIR2DL1** | 1.66E-08 | 1.132536736 | 0.06 | 0.013 | 7.46E-06 | 13 |
| **CD244** | 1.82E-06 | 1.063767551 | 0.126 | 0.05 | 0.0008151 | 13 |
| **EOMES** | 0.0001107 | 0.916359493 | 0.03 | 0.007 | 0.0497124 | 13 |
| **FOXO1** | 8.88E-05 | 0.87534057 | 0.08 | 0.031 | 0.0398656 | 13 |
| **NCAM1** | 2.47E-13 | 0.35874556 | 0.005 | 0 | 1.11E-10 | 13 |
| **CD34** | 4.74E-05 | 0.205052796 | 0.005 | 0 | 0.0212652 | 13 |
| **MMRN1** | 3.79E-07 | 0.199438968 | 0.005 | 0 | 0.00017 | 13 |
| **CCR9** | 2.50E-05 | 1.902916648 | 0.015 | 0.001 | 0.0112324 | 14 |
| **KCNE3** | 5.76E-07 | 0.965164358 | 0.007 | 0 | 0.0002588 | 14 |

**C) Classical monocytes**

| **Gene** | **p_val** | **avg_log2FC** | **pct.1** | **pct.2** | **p_val_adj** | **cluster** |
| --- | --- | --- | --- | --- | --- | --- |
| **F13A1** | 2.17E-27 | 1.0080782 | 0.212 | 0.084 | 9.72E-25 | 3 |
| **VSIG4** | 1.34E-12 | 0.9240066 | 0.054 | 0.015 | 6.02E-10 | 3 |
| **FCER2** | 1.31E-11 | 0.8471225 | 0.058 | 0.018 | 5.89E-09 | 3 |
| **MCM4** | 1.66E-06 | 0.4885603 | 0.013 | 0.002 | 0.0007468 | 3 |
| **FCGR3A** | 5.76E-33 | 2.3173304 | 0.236 | 0.088 | 2.59E-30 | 4 |
| **GZMB** | 3.60E-05 | 2.2472896 | 0.02 | 0.006 | 0.0161746 | 4 |
| **C1QA** | 2.43E-18 | 1.9128853 | 0.066 | 0.014 | 1.09E-15 | 4 |
| **IRF4** | 2.79E-05 | 1.8294686 | 0.03 | 0.01 | 0.0125302 | 4 |
| **JCHAIN** | 1.11E-09 | 1.5837504 | 0.013 | 0.001 | 4.98E-07 | 4 |
| **CD79B** | 1.10E-05 | 1.5158851 | 0.036 | 0.013 | 0.004932 | 4 |
| **CD8B** | 1.07E-06 | 1.3514541 | 0.02 | 0.004 | 0.0004805 | 4 |
| **C1QB** | 2.70E-05 | 1.2986739 | 0.024 | 0.007 | 0.0121289 | 4 |
| **IGHM-secreted** | 8.96E-05 | 1.0960307 | 0.014 | 0.003 | 0.0402492 | 4 |
| **MZB1** | 3.67E-09 | 0.7677399 | 0.009 | 0 | 1.65E-06 | 4 |
| **BLNK** | 1.82E-05 | 0.613649 | 0.011 | 0.002 | 0.0081675 | 4 |
| **BPI** | 6.78E-12 | 1.083631 | 0.049 | 0.011 | 3.04E-09 | 5 |
| **VNN2** | 1.22E-14 | 0.9969939 | 0.141 | 0.056 | 5.47E-12 | 5 |
| **AURKB** | 4.87E-05 | 0.6987883 | 0.013 | 0.002 | 0.0218747 | 5 |
| **MMP25** | 1.14E-07 | 0.5345472 | 0.058 | 0.021 | 5.11E-05 | 5 |
| **FCER1A** | 9.15E-68 | 3.1725257 | 0.145 | 0.013 | 4.11E-65 | 6 |
| **CLEC10A** | 1.65E-35 | 2.2477455 | 0.249 | 0.081 | 7.41E-33 | 6 |
| **CD1C** | 5.58E-16 | 1.9745106 | 0.119 | 0.039 | 2.51E-13 | 6 |
| **C1QA** | 1.90E-07 | 1.2546226 | 0.052 | 0.017 | 8.53E-05 | 6 |
| **C1QB** | 7.69E-05 | 1.2386709 | 0.025 | 0.007 | 0.034547 | 6 |
| **VSIG4** | 2.55E-07 | 1.1760351 | 0.052 | 0.017 | 0.0001145 | 6 |
| **LAMP3** | 9.69E-06 | 0.8604229 | 0.004 | 0 | 0.00435 | 6 |
| **CCR7** | 5.66E-06 | 0.7795829 | 0.031 | 0.009 | 0.0025413 | 6 |
| **IL1R2** | 3.44E-08 | 0.6845888 | 0.019 | 0.002 | 1.55E-05 | 6 |
| **CD2** | 0.0001098 | 0.6496317 | 0.038 | 0.014 | 0.0492882 | 6 |
| **CCL20** | 2.09E-52 | 3.8171015 | 0.5 | 0.052 | 9.39E-50 | 7 |
| **IL6** | 1.75E-58 | 3.3012142 | 0.717 | 0.097 | 7.84E-56 | 7 |
| **CD80** | 1.19E-25 | 2.9364706 | 0.15 | 0.009 | 5.33E-23 | 7 |
| **CCL4** | 3.12E-41 | 2.9030238 | 0.867 | 0.221 | 1.40E-38 | 7 |
| **CSF3** | 1.55E-20 | 2.8572021 | 0.133 | 0.009 | 6.98E-18 | 7 |
| **SOD2** | 3.96E-20 | 2.6658763 | 0.567 | 0.162 | 1.78E-17 | 7 |
| **AQP9** | 1.36E-30 | 2.6400091 | 0.683 | 0.164 | 6.10E-28 | 7 |
| **CXCL1** | 1.51E-08 | 2.2601291 | 0.233 | 0.059 | 6.76E-06 | 7 |
| **ADA** | 1.57E-13 | 1.8798151 | 0.433 | 0.122 | 7.06E-11 | 7 |
| **CD274** | 1.39E-07 | 1.6210079 | 0.033 | 0.002 | 6.24E-05 | 7 |
| **CCR10** | 8.61E-05 | 1.5105095 | 0.017 | 0.001 | 0.038671 | 7 |
| **CD28** | 8.67E-05 | 1.4027098 | 0.017 | 0.001 | 0.0389322 | 7 |
| **IGHG3-secreted** | 2.11E-07 | 0.9545728 | 0.017 | 0 | 9.46E-05 | 7 |
| **TNFRSF25** | 2.13E-07 | 0.9154655 | 0.017 | 0 | 9.57E-05 | 7 |
| **LTA** | 9.02E-06 | 0.5008227 | 0.017 | 0.001 | 0.0040479 | 7 |
| **STAT4** | 8.03E-08 | 0.430092 | 0.067 | 0.007 | 3.61E-05 | 7 |

**D) Intermediate monocytes**

| **Gene** | **p_val** | **avg_log2FC** | **pct.1** | **pct.2** | **p_val_adj** | **cluster** |
| --- | --- | --- | --- | --- | --- | --- |
| **IL3RA** | 3.67E-12 | 2.1327915 | 0.222 | 0.076 | 1.65E-09 | 1 |
| **VMO1** | 9.96E-10 | 1.6157949 | 0.146 | 0.038 | 4.47E-07 | 1 |
| **THBS1** | 1.05E-27 | 3.1269194 | 0.368 | 0.096 | 4.72E-25 | 2 |
| **S100A12** | 9.81E-38 | 2.8998061 | 0.442 | 0.103 | 4.40E-35 | 2 |
| **CD14** | 5.12E-35 | 2.3608859 | 0.537 | 0.194 | 2.30E-32 | 2 |
| **CXCL3** | 1.75E-11 | 2.1850277 | 0.221 | 0.075 | 7.87E-09 | 2 |
| **CLEC4E** | 2.94E-20 | 2.1075299 | 0.282 | 0.07 | 1.32E-17 | 2 |
| **CD163** | 1.44E-13 | 2.0568309 | 0.184 | 0.043 | 6.45E-11 | 2 |
| **CCL2** | 2.93E-07 | 1.9746691 | 0.049 | 0.003 | 0.0001317 | 2 |
| **VEGFA** | 7.84E-10 | 1.7902266 | 0.153 | 0.043 | 3.52E-07 | 2 |
| **IL1RN** | 4.25E-07 | 1.6181122 | 0.12 | 0.037 | 0.000191 | 2 |
| **PRDM1** | 1.61E-07 | 1.6001374 | 0.144 | 0.049 | 7.22E-05 | 2 |
| **MGST1** | 3.22E-10 | 1.4312623 | 0.15 | 0.039 | 1.44E-07 | 2 |
| **S100A8** | 1.90E-07 | 1.4194295 | 0.071 | 0.01 | 8.55E-05 | 2 |
| **THBD** | 5.82E-05 | 1.1239429 | 0.061 | 0.015 | 0.0261399 | 2 |

**E) Nonclassical monocytes**

| **Gene** | **p_val** | **avg_log2FC** | **pct.1** | **pct.2** | **p_val_adj** | **cluster** |
| --- | --- | --- | --- | --- | --- | --- |
| **IL3RA** | 1.68E-16 | 1.9891743 | 0.505 | 0.13 | 7.55E-14 | 3 |

**F) B cells**

| **Gene** | **p_val** | **avg_log2FC** | **pct.1** | **pct.2** | **p_val_adj** | **cluster** |
| --- | --- | --- | --- | --- | --- | --- |
| **TCL1A** | 1.38E-32 | 1.4997435 | 0.324 | 0.126 | 6.21E-30 | 1 |
| **STAT4** | 6.24E-05 | 0.7094956 | 0.031 | 0.009 | 0.0279975 | 2 |
| **CD70** | 2.41E-14 | 1.9333409 | 0.056 | 0.007 | 1.08E-11 | 4 |
| **IGHA1-secreted** | 1.61E-37 | 1.8551063 | 0.322 | 0.096 | 7.24E-35 | 4 |
| **NT5E** | 6.82E-07 | 1.0569576 | 0.064 | 0.02 | 0.0003063 | 4 |
| **IL2RA** | 8.10E-05 | 1.0448924 | 0.018 | 0.003 | 0.0363825 | 4 |
| **MGST1** | 1.48E-05 | 0.9491922 | 0.008 | 0 | 0.006656 | 4 |
| **IL3RA** | 4.74E-30 | 3.0485812 | 0.122 | 0.015 | 2.13E-27 | 5 |
| **IGHG2-secreted** | 1.17E-94 | 2.3232083 | 0.75 | 0.224 | 5.23E-92 | 6 |
| **IGHG1-secreted** | 1.15E-97 | 2.3109868 | 0.686 | 0.171 | 5.16E-95 | 6 |
| **ITGAX** | 2.97E-35 | 2.0407375 | 0.186 | 0.029 | 1.33E-32 | 6 |
| **TBX21** | 1.74E-12 | 1.8476644 | 0.058 | 0.008 | 7.79E-10 | 6 |
| **LGALS1** | 9.68E-29 | 1.6464804 | 0.426 | 0.168 | 4.35E-26 | 6 |
| **IGHG3-secreted** | 2.86E-14 | 1.6177873 | 0.125 | 0.033 | 1.28E-11 | 6 |
| **SRGN** | 1.03E-08 | 1.5762712 | 0.08 | 0.023 | 4.62E-06 | 6 |
| **IGHG4-secreted** | 1.57E-09 | 1.5319503 | 0.071 | 0.017 | 7.04E-07 | 6 |
| **FCGR2A** | 4.76E-19 | 1.5200648 | 0.103 | 0.017 | 2.14E-16 | 6 |
| **IGHG1-membrane** | 5.87E-09 | 1.5061436 | 0.058 | 0.012 | 2.63E-06 | 6 |
| **HOPX** | 1.08E-10 | 1.5010793 | 0.147 | 0.054 | 4.86E-08 | 6 |
| **CST7** | 6.33E-09 | 1.4641855 | 0.074 | 0.019 | 2.84E-06 | 6 |
| **FCER1A** | 5.78E-05 | 1.3676275 | 0.006 | 0 | 0.0259663 | 6 |
| **NEAT1** | 4.96E-12 | 1.2219742 | 0.192 | 0.075 | 2.23E-09 | 6 |
| **CD27** | 5.17E-07 | 1.0161689 | 0.09 | 0.032 | 0.0002322 | 6 |
| **AIM2** | 1.73E-07 | 1.0020906 | 0.061 | 0.016 | 7.75E-05 | 6 |
| **CD86** | 9.23E-07 | 0.9401141 | 0.106 | 0.042 | 0.0004146 | 6 |
| **SLC7A7** | 9.41E-05 | 0.8601451 | 0.051 | 0.017 | 0.0422331 | 6 |
| **IL2RB** | 3.75E-05 | 0.493348 | 0.042 | 0.011 | 0.0168503 | 6 |
| **CCR1** | 5.78E-05 | 0.3804013 | 0.006 | 0 | 0.0259663 | 6 |
| **CD27** | 4.88E-12 | 1.7999202 | 0.124 | 0.031 | 2.19E-09 | 7 |
| **CXCR3** | 1.14E-11 | 1.5334383 | 0.064 | 0.009 | 5.14E-09 | 7 |
| **AIM2** | 1.69E-08 | 1.4691201 | 0.073 | 0.016 | 7.57E-06 | 7 |
| **IGHG1-secreted** | 4.21E-42 | 1.4572799 | 0.606 | 0.196 | 1.89E-39 | 7 |
| **TCF7** | 5.33E-08 | 1.4366394 | 0.055 | 0.01 | 2.39E-05 | 7 |
| **NCR3** | 4.23E-16 | 1.4348106 | 0.188 | 0.05 | 1.90E-13 | 7 |
| **IGHG2-secreted** | 2.27E-44 | 1.4240544 | 0.693 | 0.248 | 1.02E-41 | 7 |
| **IGHG4-secreted** | 1.81E-05 | 1.4117447 | 0.064 | 0.019 | 0.0081075 | 7 |
| **CD86** | 3.60E-12 | 1.353479 | 0.147 | 0.041 | 1.62E-09 | 7 |
| **IGHA1-secreted** | 8.33E-32 | 1.2886714 | 0.39 | 0.105 | 3.74E-29 | 7 |
| **IGHG3-secreted** | 5.76E-08 | 1.14885 | 0.115 | 0.037 | 2.59E-05 | 7 |
| **CTSA** | 4.10E-08 | 1.0120381 | 0.151 | 0.056 | 1.84E-05 | 7 |

**G) NK cells**

| **Gene** | **p_val** | **avg_log2FC** | **pct.1** | **pct.2** | **p_val_adj** | **cluster** |
| --- | --- | --- | --- | --- | --- | --- |
| **LAG3** | 1.21E-10 | 1.263858 | 0.088 | 0.02 | 5.45E-08 | 1 |
| **SPON2** | 9.81E-08 | 1.4836337 | 0.055 | 0.012 | 4.40E-05 | 2 |
| **ZBTB16** | 2.25E-07 | 1.1941327 | 0.08 | 0.026 | 0.0001011 | 2 |
| **CXCR2** | 3.39E-05 | 1.02881 | 0.027 | 0.004 | 0.0152194 | 2 |
| **IL7R** | 2.19E-56 | 3.0306981 | 0.256 | 0.014 | 9.82E-54 | 3 |
| **GZMK** | 5.58E-73 | 3.022682 | 0.419 | 0.037 | 2.50E-70 | 3 |
| **SELL** | 1.42E-50 | 2.5255503 | 0.57 | 0.133 | 6.36E-48 | 3 |
| **LEF1** | 2.26E-31 | 2.310952 | 0.203 | 0.021 | 1.01E-28 | 3 |
| **IL18R1** | 5.25E-31 | 2.1080797 | 0.233 | 0.029 | 2.36E-28 | 3 |
| **ITGAX** | 8.18E-23 | 1.6871465 | 0.506 | 0.188 | 3.67E-20 | 3 |
| **STAT1** | 3.34E-09 | 1.4541919 | 0.11 | 0.025 | 1.50E-06 | 3 |
| **CD44** | 7.73E-19 | 1.4008855 | 0.384 | 0.123 | 3.47E-16 | 3 |
| **TCF7** | 3.12E-10 | 1.3329839 | 0.105 | 0.02 | 1.40E-07 | 3 |
| **MYC** | 5.14E-08 | 1.3240981 | 0.087 | 0.018 | 2.31E-05 | 3 |
| **CXCR3** | 1.05E-07 | 1.3132578 | 0.116 | 0.032 | 4.70E-05 | 3 |
| **TNFSF10** | 1.83E-08 | 1.2983334 | 0.122 | 0.032 | 8.20E-06 | 3 |
| **CD300A** | 6.71E-26 | 1.2846343 | 0.5 | 0.158 | 3.01E-23 | 3 |
| **IL12RB2** | 1.93E-10 | 1.247813 | 0.052 | 0.004 | 8.65E-08 | 3 |
| **VEGFA** | 8.44E-06 | 1.2254885 | 0.017 | 0.001 | 0.0037898 | 3 |
| **ICAM1** | 8.12E-06 | 0.9648759 | 0.151 | 0.058 | 0.003645 | 3 |
| **IL18RAP** | 8.58E-12 | 0.8928183 | 0.314 | 0.115 | 3.85E-09 | 3 |
| **IL4R** | 2.22E-06 | 0.8069823 | 0.163 | 0.062 | 0.0009973 | 3 |
| **TIAF1** | 9.16E-06 | 0.7819986 | 0.134 | 0.049 | 0.0041146 | 3 |
| **CD79B** | 2.27E-05 | 0.7620078 | 0.035 | 0.005 | 0.0101796 | 3 |

**Table S8. Significantly differentially expressed genes for each cell type (A-E).** Differentially expressed genes are from one cluster compared against the rest in the same cell type. gene: gene name, p_val: raw p value, avg_logFC: average log2 fold change, pct.1: percent of cells in hat cluster that express this gene, pct.2: percent of cells in all other clusters that express this gene, p_val_adj: p value adjusted by Benjamini-Hochberg for multiple comparisons, cluster: cluster number as noted in UMAP. Significant genes were defined as adjusted p<0.01, avg_locFC>0, and pct1/pct2>0.2

Table S9.

| **Patient number** | **Pat_Type** | **Total no. of cells** | **B cells** | **CD4 T cells** | **CD8 T cells** | **Monocytes** | **NK cells** |
| --- | --- | --- | --- | --- | --- | --- | --- |
| 1 | HIV+CVD+CRT+ | 1,658 | 29 | 310 | 754 | 129 | 37 |
| 2 | HIV+CVD+CRT- | 1,869 | 322 | 791 | 424 | 263 | 24 |
| 3 | HIV+CVD-CRT- | 1,799 | 115 | 275 | 1,067 | 151 | 90 |
| 4 | HIV-CVD-CRT- | 1,164 | 111 | 615 | 294 | 115 | 13 |
| 5 | HIV+CVD+CRT+ | 358 | 22 | 91 | 160 | 11 | 28 |
| 7 | HIV+CVD-CRT- | 2,574 | 102 | 602 | 757 | 683 | 226 |
| 8 | HIV-CVD-CRT- | 394 | 39 | 152 | 63 | 91 | 45 |
| 9 | HIV+CVD+CRT+ | 708 | 98 | 208 | 215 | 93 | 59 |
| 10 | HIV+CVD+CRT- | 951 | 86 | 291 | 428 | 109 | 31 |
| 11 | HIV+CVD-CRT- | 514 | 31 | 140 | 127 | 179 | 18 |
| 12 | HIV-CVD-CRT- | 675 | 22 | 292 | 150 | 158 | 8 |
| 13 | HIV+CVD+CRT+ | 964 | 45 | 185 | 360 | 197 | 94 |
| 14 | HIV+CVD+CRT- | 737 | 49 | 71 | 431 | 113 | 16 |
| 15 | HIV+CVD-CRT- | 1,366 | 41 | 205 | 264 | 788 | 34 |
| 16 | HIV-CVD-CRT- | 1,266 | 60 | 696 | 254 | 150 | 84 |
| 17 | HIV+CVD+CRT+ | 748 | 90 | 202 | 235 | 161 | 36 |
| 18 | HIV+CVD+CRT- | 1,178 | 66 | 414 | 488 | 144 | 30 |
| 19 | HIV+CVD-CRT- | 1,323 | 98 | 526 | 429 | 195 | 38 |
| 20 | HIV-CVD-CRT- | 1,244 | 66 | 799 | 161 | 150 | 52 |
| 21 | HIV+CVD+CRT+ | 973 | 144 | 253 | 264 | 226 | 59 |
| 22 | HIV+CVD+CRT- | 1,836 | 152 | 178 | 707 | 455 | 60 |
| 23 | HIV+CVD-CRT- | 958 | 49 | 77 | 413 | 203 | 141 |
| 24 | HIV-CVD-CRT- | 1,094 | 67 | 488 | 218 | 168 | 21 |
| 25 | HIV+CVD+CRT+ | 1,436 | 160 | 557 | 250 | 365 | 68 |
| 26 | HIV+CVD+CRT- | 1,141 | 70 | 289 | 436 | 95 | 173 |
| 27 | HIV+CVD-CRT- | 766 | 112 | 348 | 183 | 101 | 16 |
| 28 | HIV-CVD-CRT- | 964 | 70 | 425 | 190 | 50 | 49 |
| 29 | HIV+CVD+CRT+ | 1,017 | 43 | 218 | 310 | 114 | 38 |
| 30 | HIV+CVD+CRT- | 1,117 | 213 | 299 | 190 | 75 | 137 |
| 31 | HIV+CVD-CRT- | 961 | 100 | 350 | 228 | 87 | 47 |
| 32 | HIV-CVD-CRT- | 1,526 | 163 | 672 | 415 | 53 | 29 |

**Table S9: Number of Cells for each participant.** Number of cells for each Patient in our dataset. Patient 6 has no cells. The column Pat_Type gives the type of the patient. “HIV+” denotes patients with HIV, “CVD+” denotes patients with cardiovascular disease, “CRT+” denotes patients with cholesterol reduce treatment, “HIV-” denotes patients without HIV, “CVD-” denotes patients without cardiovascular disease and “CRT-” denotes patients without cholesterol reduce treatment.

**Table S10**

| **Gene** | **Antibody** | | **CD4T** | **CD8T** | **CM** | **INT** | **NCM** | **B** | **NK** |
| --- | --- | --- | --- | --- | --- | --- | --- | --- | --- |
| *ITGAM* | CD11b | 0.0518 | | 0.0098 | 0 | 0 | 0 | 0 | 0 |
| *ITGAX* | CD11c | 0.0138 | | 0.0514 | 0 | 0 | 0.0119 | 0 | 0 |
| *IL3RA* | CD123 | 0.0133 | | 0.0052 | 0.0252 | 0.0113 | 0 | 0 | 0.0624 |
| *IL7R* | CD127 | 0 | | 0 | 0 | 0.0218 | 0.0188 | 0.0022 | 0 |
| *THBD* | CD141 | 0.0118 | | 0.0033 | 0.0086 | 0 | 0 | 0 | 0.0076 |
| *CD14* | CD14 | 0.0205 | | 0.0089 | 0 | 0 | 0.0151 | 0 | 0.0277 |
| *CTLA4* | CD152 | 0.0593 | | 0.0240 | 0 | 0 | 0 | 0 | 0 |
| *CD163* | CD163 | 0.0113 | | 0 | 0 | 0 | 0.0144 | 0.0028 | 0 |
| *FCGR3A* | CD16 | 0.0419 | | 0.0555 | 0.1488 | 0.0038 | 0 | 0 | 0 |
| *CXCR3* | CD183 | 0.0274 | | 0.0304 | 0 | 0 | 0 | 0 | 0.0259 |
| *CXCR4* | CD184 | 0 | | 0 | 0.0228 | 0.0542 | 0 | 0.0792 | 0 |
| *CXCR5* | CD185 | 0 | | 0.0035 | 0.0543 | 0.0208 | 0 | 0.0038 | 0 |
| *CCR2* | CD192 | 0 | | 0.0201 | 0 | 0 | 0 | 0.0317 | 0 |
| *CCR4* | CD194 | 0.0115 | | 0 | 0.0069 | 0 | 0 | 0.0099 | 0 |
| *CCR5* | CD195 | 0.0089 | | 0.0551 | 0.0333 | 0.0350 | 0 | 0 | 0.0442 |
| *CCR6* | CD196 | 0 | | 0 | 0.0137 | 0 | 0 | 0.0221 | 0 |
| *CCR7* | CD197 | 0 | | 0 | 0.0911 | 0.0294 | 0 | 0.0810 | 0 |
| *MS4A1* | CD20 | 0.0240 | | 0 | 0 | 0.0337 | 0 | 0 | 0.0533 |
| *LAG3* | CD223 | 0.0421 | | 0.0539 | 0 | 0.0195 | 0 | 0.0012 | 0.0813 |
| *IL2RA* | CD25 | 0 | | 0 | 0.0110 | 0.0104 | 0 | 0 | 0.0110 |
| *CD27* | CD27 | 0 | | 0 | 0 | 0 | 0 | 0 | 0 |
| *CD2* | CD2 | 0.0058 | | 0 | 0.0278 | 0 | 0 | 0 | 0.0541 |
| *CD36* | CD36 | 0.0120 | | 0.0042 | 0 | 0 | 0.0957 | 0 | 0 |
| *CD38* | CD38 | 0 | | 0.0347 | 0 | 0.0466 | 0 | 0 | 0 |
| *CD3D* | CD3 | 0 | | 0 | 0 | 0 | 0 | 0 | 0 |
| *CD3E* | CD3 | 0 | | 0 | 0.0011 | 0 | 0 | 0 | 0.1495 |
| *CD3G* | CD3 | 0.0069 | | 0.0077 | 0 | 0 | 0.0519 | 0.0142 | 0.0237 |
| *PTPRC* | CD45RA | 0 | | 0 | 0 | 0 | 0 | 0 | 0.0164 |
| *PTPRC* | CD45RO | 0 | | 0 | 0 | 0 | 0 | 0 | 0.0164 |
| *CD4* | CD4 | 0.0030 | | 0 | 0 | 0 | 0 | 0 | 0.0289 |
| *NCAM1* | CD56 | 0.0275 | | 0.0083 | 0 | 0 | 0 | 0 | 0.0030 |
| *CD69* | CD69 | 0 | | 0 | 0.0293 | 0 | 0 | 0.0909 | 0 |
| *CD86* | CD86 | 0.0140 | | 0.0176 | 0.0391 | 0.0174 | 0.0151 | 0 | 0.0400 |
| *CD8A* | CD8 | 0.0049 | | 0.0185 | 0 | 0.0158 | 0 | 0 | 0 |
| *CD8B* | CD8 | 0.0102 | | 0 | 0.0022 | 0 | 0.0489 | 0 | 0.0352 |
| *CD9* | CD9 | 0.0278 | | 0 | 0.0315 | 0 | 0 | 0.0104 | 0.0028 |
| *CD74* | HLA | 0.2211 | | 0.3454 | 0.3687 | 0.4684 | 0.3321 | 0.1147 | 0.3648 |
| *HLA* | HLA | 0.0412 | | 0 | 0.0115 | 0.0043 | 0.1062 | 0 | 0 |

**Table S10. Non-negative Spearman correlation between antibody and gene in each cell type.** All; all cell types, CM; Classical monocytes, INT; intermediate monocytes, NCM; Nonclassical monocytes. Dense color indicates higher value.

Additional file 2: Data S1. (separate excel file). Data underlying Figure 5, dotplots of DEGs for disease types. Dotplots of differentially expressed genes between HIV+CVD-CRT- vs HIV-CVD-CRT-, between HIV+CVD+CRT- vs HIV+CVD-CRT-, and between HIV+CVD+CRT+ vs HIV+CVD+CRT- in each cell type

**Additional file 3: Data S2 (separate excel file). Data underlying Figure 6, random forest model.** The feature importance of each gene in the comparison between non-HIV vs HIV, HIV+CVD- vs HIV+CVD+ and HIV+CVD+CRT- vs HIV+CVD+CRT+.
